# ICON-GEMs: integration of co-expression network in genome-scale metabolic models, shedding light through systems biology

**DOI:** 10.1186/s12859-023-05599-0

**Published:** 2023-12-21

**Authors:** Thummarat Paklao, Apichat Suratanee, Kitiporn Plaimas

**Affiliations:** 1 Advanced Virtual and Intelligent Computing (AVIC) Center, Department of Mathematics and Computer Science, Faculty of Science, Chulalongkorn University, Bangkok, 10330 Thailand; 2 Department of Mathematics, Faculty of Applied Science, King Mongkut’s University of Technology North Bangkok, Bangkok, 10800 Thailand; 3 Omics Sciences and Bioinformatics Center, Faculty of Science, Chulalongkorn University, Bangkok, 10330 Thailand

**Keywords:** Flux balance analysis (FBA), Constraint-based approach, Gene co-expression network, *Escherichia coli*, *Saccharomyces cerevisiae*, Quadratic programming, Metabolic flux analysis, Metabolic engineering, Transcriptomic data, Genome-scale metabolic model

## Abstract

**Background:**

Flux Balance Analysis (FBA) is a key metabolic modeling method used to simulate cellular metabolism under steady-state conditions. Its simplicity and versatility have led to various strategies incorporating transcriptomic and proteomic data into FBA, successfully predicting flux distribution and phenotypic results. However, despite these advances, the untapped potential lies in leveraging gene-related connections like co-expression patterns for valuable insights.

**Results:**

To fill this gap, we introduce ICON-GEMs, an innovative constraint-based model to incorporate gene co-expression network into the FBA model, facilitating more precise determination of flux distributions and functional pathways. In this study, transcriptomic data from both *Escherichia coli* and *Saccharomyces cerevisiae* were integrated into their respective genome-scale metabolic models. A comprehensive gene co-expression network was constructed as a global view of metabolic mechanism of the cell. By leveraging quadratic programming, we maximized the alignment between pairs of reaction fluxes and the correlation of their corresponding genes in the co-expression network. The outcomes notably demonstrated that ICON-GEMs outperformed existing methodologies in predictive accuracy. Flux variabilities over subsystems and functional modules also demonstrate promising results. Furthermore, a comparison involving different types of biological networks, including protein–protein interactions and random networks, reveals insights into the utilization of the co-expression network in genome-scale metabolic engineering.

**Conclusion:**

ICON-GEMs introduce an innovative constrained model capable of simultaneous integration of gene co-expression networks, ready for board application across diverse transcriptomic data sets and multiple organisms. It is freely available as open-source at https://github.com/ThummaratPaklao/ICOM-GEMs.git.

**Supplementary Information:**

The online version contains supplementary material available at 10.1186/s12859-023-05599-0.

## Background

Exploring an organism's phenotypes involves various aspects like growth rates, reaction rates, and production rates [1], with broad applications in fields like metabolic engineering, agriculture, and biotechnology [2–6]. Phenotypes stem from genotypes, where gene expression, a complex process, shapes an organism's traits and associations among genes, impacting reaction rates and fluxes. Predicting flux distribution at a state-specific level enhances the understanding of cellular metabolism's functional states [7]. While experimental and computational methods are used to deduce reaction fluxes, they each come with limitations – experimental methods like 13C metabolic flux analysis require specialized expertise and instrumentation, while computational methods face challenges in data comprehensiveness and algorithm design [8–10]. Genome-scale metabolic models (GEMs) are vital in silico tools for understanding cellular behavior and predicting reactions, genes, and responses to the environment [11, 12]. GEMs represent the relationships among metabolites, reactions, and genes in organisms, finding diverse applications from metabolic engineering to disease insights [13].

Various mathematical tools estimate metabolic flow and reaction fluxes in organisms via genome-scale metabolic networks using optimization, differential equations, and stochastic simulations. Among these, flux balance analysis (FBA) [14–16] stands out, optimizing growth rates and production under steady-state constraints. While FBA is effective, predicting flux distribution isn't infallible due to multiple potential solutions, prompting the need to refine solutions by introducing context-specific constraints and optimizing objective functions [17, 18].

Utilizing precious transcriptomic data, GEMs coupled with FBA have evolved to enhance flux distribution predictions. This enhancement is achieved as assimilating transcriptomic data into metabolic models through gene-protein-reaction (GPR) associations [18–22]. Transcriptomic data provides a powerful constraint on potential solutions in genome-scale metabolic models (GEMs). There are two main categories for integrating transcriptomic data into GEMs. The first category involves binary gene expression states, requiring threshold, as seen in methods like Gene Inactivity Moderated by Metabolism and Expression (GIMME) [23] and ensures a functional model while quantifying consistency with expression data. Jensen and Papin introduced an approach named Metabolic Adjustment by Differential Expression (MADE), which does not rely on arbitrary thresholding [24]. MADE quantifies the meaningful variations in gene expression data. The second category directly integrates expression data, such as the E-flux method [25], which assigns reaction flux bounds based on measured gene expression, leading to more precise predictions. Kim et al. [21] introduced E-flux2 and SPOT in a dual-method approach to handle scenarios with unknown biological objectives. Importantly, these techniques exclusively use gene expression values.

Various methods, such as LBFBA [26], ETFL [27], REMI [28], and DeltaFBA [29], leverage diverse data types to enhance Flux Balance Analysis. TRFBA [30], which focuses on gene interactions within transcriptional regulatory networks, has limitations due to the availability of complete networks, mainly applicable to select organisms.

Furthermore, cooperation among genes can be unveiled through analysis of gene expressions using co-expression networks. In general, gene co-expression networks form gene–gene association networks by calculating pairwise gene similarity scores from gene expression levels [31]. These networks are typically represented as undirected graphs with a designated threshold indicating gene relationships. Strong connection exists between genes with high correlations. Gene co-expression network analysis serves various purposes, including identifying functionally related genes and co-regulated genes by common transcriptional factors. Additionally, gene co-expression networks reveal cooperative relationships, offering insights into functional modules within cells. Integrating these networks into GEMs holds promise for further advancements.

In this study, we propose a quadratic programming model to integrate the gene co-expression network instead of using solely the expression values to GEMs. The objective is to enhance the quantitative and qualitative simulation and predicting of condition-specific metabolic networks based on gene expression patterns. This approach facilitates a more precise identification of functional modules involved in metabolic processes under specific circumstances. We applied our proposed model to the GEMs of *Escherichia coli* (*E. coli*) and *Saccharomyces cerevisiae* (*S. cerevisiae*), followed by a comprehensive comparative analysis of the outcomes and findings against existing methodologies employing diverse strategies.

## Materials and methods

### Workflow of integrating gene co-expression network and metabolic model

**Fig. 1:**
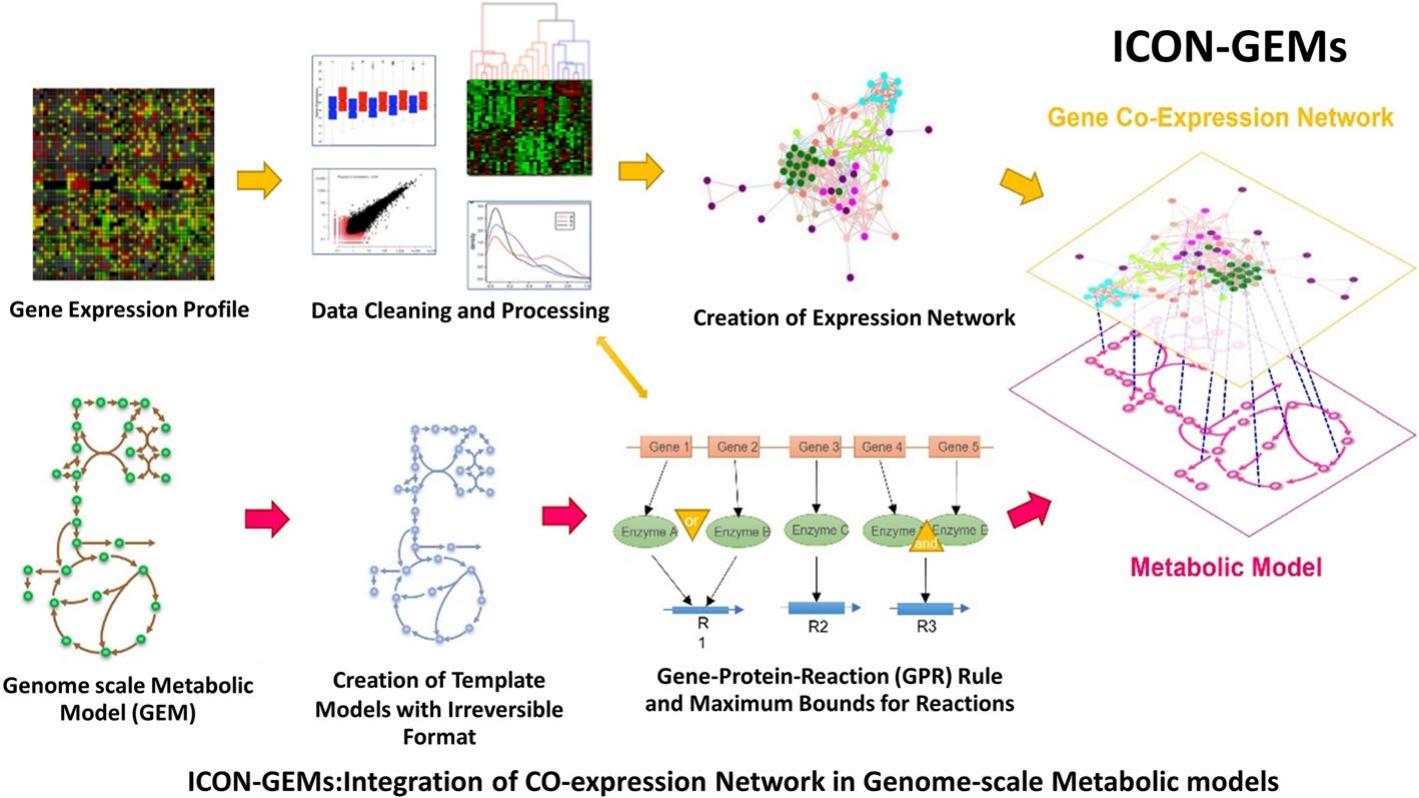
Workflow diagram representing the process of ICON-GEMs to integrate a gene co-expression network into a genome-scale metabolic model. It starts with gene expression profiles and genome-scale metabolic models. Data cleaning and processing are applied to the gene expression data to construct a gene co-expression network. The template model is constructed, and gene-protein-reaction (GPR) rule is used to quantify the associated reactions. Finally, a gene co-expression network and a metabolic network are merged for calculating flux rates in ICON-GEMs formulation

Our workflow of integrating co-expression network and metabolic model is illustrated in Fig. 1. The workflow began with the preparation of gene expression profiles, involving the handling of missing values and outliers. Subsequently, these prepared profiles were utilized to construct the co-expression network, where Pearson correlations were employed to calculate correlation coefficients. These coefficients were then transformed into a binary adjacency matrix. Similarly, the genome-scale metabolic model was prepared through the creation of a template metabolic model, with reaction flux bounds set without uptake rate information. The originally reversible reaction fluxes within the model were transformed into irreversible reaction fluxes. To integrate gene expression data for each condition into the metabolic model, additional constraints to limit reaction fluxes in the same manner as the E-flux method and the gene co-expression network were combined into a quadratic programming model, named as ICON-GEMs. Note that ICON-GEMs requires four inputs: (1) a gene expression profile, (2) a genome-scale metabolic model, (3) specific conditions for flux distribution calculations, and (4) a threshold to differentiate high and low correlation for creating a gene co-expression network.

### The formulation of ICON-GEMs

ICON-GEMs utilize quadratic programming to integrate a gene co-expression network into a metabolic network based on flux balance analysis (FBA). This integration relies on the principle that when a pair of genes exhibits high correlation, the corresponding reactions are also correlated. The objective of this quadratic programming is to optimize the amalgamation of correlation between flux-carrying pairs reactions. In essence, ICON-GEMs seek to maximize the sum of products of transformed flux values for reaction pairs corresponding to any two genes connected in the co-expression network. In the ICON-GEMs context, the E-flux technique [25] is applied to establish the upper bounds for flux values in FBA, contingent on measured gene expression levels, using gene-protein-reaction (GPR) association [22]. In irreversible models, it's common to establish lower flux bounds at zero. The quadratic programming model within ICON-GEMs can be formulated as follows:

1 $${\text{Maximize}}\mathop \sum \limits_{{\left( {i,j} \right) \in R}} q_{i} q_{j}$$

2 $${\text{Subject to }}\mathop \sum \limits_{j = 1}^{n + p} \overline{S}_{ij} \overline{v}_{j} = 0$$

3 $$0 \le \overline{v}_{j} \le f\left( {g_{j} } \right)\;{\text{for all }}j = 1, 2, 3, \ldots , n + p$$

4 $$\mathop \sum \limits_{j = 1}^{n + p} \overline{c}_{j} \overline{v}_{j} \ge \alpha z^{*}$$

5 $$\mathop \sum \limits_{{\left( {i,j} \right) \in Rev}} \overline{v}_{i} \overline{v}_{j} = 0$$

6 $$\frac{{\overline{v}_{j} }}{{M_{j} }} - q_{j} = - 1\; {\text{for all }}j = 1, 2, 3, \ldots , n + p$$

where $q_{i} {\text{ and }}q_{j}$ represent transformed reaction flux values of reaction *i* and *j*, respectively, while $R$ is the set of reaction pairs whose genes are linked in the co-expression network. It is worth noting that the objective function in Eq. (1) is a summation of the product $q_{i} q_{j}$, specifically for reactions *i* and *j* that correspond to genes connected in the co-expression network. $$\overline{S} = \left[ {\begin{array}{*{20}c} {S^{irr} } & {S^{rev} } & {S^{rev} } \\ \end{array} } \right]^{T}$$ comprises submatrices $S^{irr}$ and $S^{rev}$, which pertain to columns of $S$ corresponding irreversible and reversible reaction fluxes, respectively. $$\overline{v} = \left[ {\begin{array}{*{20}c} {v^{irr} } & {v^{rev} } & { - v^{rev} } \\ \end{array} } \right]^{T}$$ is a vector comprising irreversibly oriented and reversibly oriented flux components, with $v^{rev}$ signifying the reversible component. $f\left( {g_{j} } \right)$ is a function to transform a gene expression value $g_{j}$ to a related flux bound value. In our study, we define $f\left( {g_{j} } \right) = g_{j}$. $$\overline{c} = \left[ {\begin{array}{*{20}c} {c^{irr} } & {c^{rev} } & {c^{rev} } \\ \end{array} } \right]^{T}$$ is a vector encompassing irreversible and reversible reaction flux components, where $c^{irr}$ and $c^{rev}$ correspond to irreversible and reversible fluxes, respectively. The $\overline{c}$ is a vector of zeros with a one at the position of the reaction of interest (biomass flux). $z^{*}$ denotes the potential maximum biomass, predicted through the E-flux method [25]. $\alpha \in \left( {0,1} \right]$ is used to determine the proportion of biomass required to ascertain the vitality of organisms. In our study the value of $\alpha$ is set to 1. $Rev$ represents a set of reaction pairs derived from the same reversible reaction flux. $M_{j}$ signifies the maximum gene expression value for reaction flux $j$.

Constraints (2)-(3) mirror those of the E-flux model [25]. Constraint (4) establishes the biomass value as the maximum attainable value, denoted by $z^{*}$, in E-flux. Constraint (5) involves the summation of products of irreversible reaction flux pairs derived from the same reversible reaction flux, where $Rev$ comprises these pairs. Additionally, Constraint (6) represents a modified equation. More comprehensive and detailed explanations of our ICON-GEMs formulation can be found in Additional file 1.

### Construction of a template metabolic model

We have developed an integrated model using gene expression data and the genome scale-metabolic model. However, there is inconsistency between the units used to measure metabolic reaction flux and the those used for gene expression. Therefore, we construct a template metabolic model to avoid this problem, following the approach outlined in [21]. The template metabolic model retains the stoichiometric and reversibility information while discarding the specific flux rate constraints present in the original genome-scale metabolic model. A way to construct the template model is to set the flux bounds of each reaction to either zero or to the largest absolute value, denoted by $T$. Noted that *T* is a variable standing for the largest number or maximum values for the model calculation. It is not a threshold for setting the uptake rate of any carbon sources.

Suppose that there are $m$ metabolites and $n$ reactions in a metabolic network. Let $\hat{L}$ and $\hat{U}$ be the new lower and upper bound of flux of reaction in template metabolic model, respectively:

7 $$ \hat{L}_{j} = \left\{ {\begin{array}{*{20}c} {\;\;\;0 \;\;\;\;\text{if}\; L_{j} \ge 0} \\ { - T \;\;\;\text{if}\; L_{j} < 0} \\ \end{array} } \right. \text{and }\;\hat{U}_{j} = \left\{ {\begin{array}{*{20}c} {T \;\;\;\;\text{if}\; U_{j} > 0} \\ {0 \;\;\;\;\text{if}\; U_{j} \le 0} \\ \end{array} } \right. {\text{for all }}j = 1,2,3,...,n$$

In some situations, the used carbon sources in the cell are unknown. Thus, this template metabolic model is constructed in two different cases depending on the information of carbon source. The first template metabolic model, known as the DC (Determined Carbon Source) model, sets the lower bound of known carbon source reactions to a negative value of the largest number. The known carbon source for the DC model is glucose, as the experimental data used to demonstrate it in this study were measured by feeding glucose into the system. The second model is known as the AC (All Possible Carbon Source) model, which sets a negative value representing the largest number as the lower bound for the fluxes of all carbon source reactions in the metabolic model.

### Flexibility of flux reactions and subsystems

Given that alternative optimal solutions may exist within various constraint-based methods, the flexibility of flux is employed to explore alternative solutions or the range of potential reaction fluxes within a metabolic system. We employ a two-stage programming approach known as Flux Variability Analysis (FVA) [32] to assess the flexibility of flux reactions. In the first stage programming, a relevant objective function, denoted as $Z^{*}$, is computed by applying ICON-GEM. Subsequently, the second stage employs a constraint-based modeling technique to evaluate the minimum and maximum range of each reaction flux in the metabolic model while still yielding a well-defined value of the objective function from the first-stage programming. In the second stage, the objective function is designed to find the maximum and minimum flux values of each reaction. Furthermore, the objective function employed in the first stage serves as an additional constraint to fulfill the requirements of $Z^{*}$. Assume that, for reaction *i*, we have obtained the maximum flux value ($v_{max}^{i}$) and the minimum flux value $(v_{min}^{i}$) through FVA. We determine the flexibility of reaction $i$ ($F_{i}$) using the following formula:

8 $$F_{i} = \left| {v_{max}^{i} - v_{min}^{i} } \right|.$$

The flexibility of each reaction in the model has been computed across all subsystems defined in the genome-scale metabolic models of both *E. coli* and *S. cerevisiae*. Within each subsystem, flexibility values for individual reactions, ($F_{i}$) as defined in Eq. (8), are determined. These individual flexibility values for reactions within the same subsystem are then averaged to assess the overall flexibility of that subsystem.

This method provides valuable insights into the behavior of individual reactions within each subsystem. It facilitates the identification of reactions that display high flexibility, enabling them to adapt to varying conditions, as well as reactions that exhibit greater rigidity in their flux. This analysis contributes to a better understanding of how different components within the subsystem interact and contribute to the overall metabolic function.

### Gene co-expression network

A gene co-expression network serves as an illustrative framework to depict gene cooperation, relying on the correlation coefficient [31]. This network takes the form of an undirected graph, with nodes symbolizing genes translated from transcriptomic data to genome-scale metabolic model. Connections between nodes signify correlations between pairs of genes. Let $A$ be an adjacency, a symmetric array. The entries within this matrix quantify the strength of connections between gene pairs. The process of constructing a co-expression network comprises two steps. Firstly, the identification and removal of missing values and outliers are undertaken. Subsequently, the Pearson correlation calculates gene correlations, followed by the application of a thresholding method to transform the correlation matrix into a binary matrix. The determination of a suitable threshold involves the consideration of various factors, including scale-free topology, mean connectivity, and cluster count, as elucidated in [33]. This process of selection entails choosing the threshold that maximizes cluster count while maintaining compliance with the scale-free topology criteria (with an $R^{2}$ value of at least 0.5). Correlation coefficients falling below a defined threshold are set to zero, while those surpassing the threshold are set to one. This process ensures the generation of a coherent co-expression network.

### Predictive accuracy measurement

The predictive accuracy of our algorithm was evaluated using the uncentered Pearson product-moment correlation between the predicted fluxes and the measured fluxes obtained through 13C-metabolic flux analysis. Utilizing uncentered Pearson product moment correlation to compare measured and predicted fluxes serves two primary reasons: overcoming the issue of differing units between predicted and measured fluxes and focusing on capturing the linear relationships between the patterns or trends in the flux data. It is important to note that the correspondence between measured and predicted fluxes is not straightforward due to the intricate interplay of genes, proteins, reactions, and metabolites within a metabolic model. Consequently, a direct one-to-one mapping of predicted to measured fluxes is not applicable.

To address this complexity, we employed both "OR" and "AND" relationships for the reaction fluxes related to the substances and the products of the measured reaction fluxes. There are two possible scenarios for mapping the predicted fluxes to the experimental measurements, as follows:

*Case 1*: When multiple reaction steps are required to produce the same desired products as the measured reaction flux. This means that to go from the substrates of the actual measured reaction to the products, several intermediate reactions and metabolites need to be involved through various modeled reactions. For instance, if the experiment measures the flux of a true reaction from A to D, but in the metabolic model, going from substrate A to product D involves multiple sequential reactions (A → B, B → C, and then C → D), we used an "AND" relationship to calculate a predicted flux for this measured flux. This calculation involved determining the minimum flux value found in the chain of reactions from A to D within the metabolic model.

*Case 2*: In situations where multiple reactions involve the same sets of metabolites as those associated with the measured flux, we utilized the total sum of the predicted fluxes in the metabolic model for comparison with the measured fluxes. This established an "OR" relationship among modeled reactions sharing identical substrates and products. For instance, if the experiment measures the flux of reaction A → D, and within the model, there exist three possible reactions that both consume A and produce D, such as A + B → C + D, A → D + E, and A + F → D, to represent the predicted flux for the reaction A → D, we calculated the cumulative sum of the predicted fluxes for these three modeled reactions, which was then compared with the measured flux.

After the conversion and mapping between measured fluxes and predicted fluxes, we measure the predictive accuracy as the similarity between those fluxes is calculated based on the uncentered Pearson product moment correlation, denoted as *R*, which can be computed as follows:

9 $$R = \frac{{v_{p}^{T} v_{m} }}{{v_{p} v_{m} }}$$

where $v_{p}$ and $v_{m}$ represent the vectors of predicted and measured fluxes, respectively. The value of this correlation coefficient near + 1 or -1 indicates a strong positive or negative linear relationship between $v_{p}$ and $v_{m}$. Conversely, a correlation coefficient close to 0 implies the absence of a linear relationship between the two vectors. For more detailed on the mapping and the calculation can be found in Additional file 1.

### Software availability

The proposed method of ICON-GEMs was implemented using MATLAB version 2018a and is available on GitHub at https://github.com/ThummaratPaklao/ICOM-GEMs.git [34]. This program necessitates the use of the COBRA toolbox [35, 36] as well as a quadratic solver provided by Gurobi Optimizer (version 9.0) [37].

### Expression datasets and genome-scale metabolic models

We validate and test our ICON-GEMs with the transcriptomic data and genome-scale metabolic models of *E. coli* and *S. cerevisiae*.

For *E. coli*, we utilized the transcriptomic data from the study of Ishii et al. [38],which provided both gene expression and 13C metabolic flux data under the same conditions. This dataset consisted of 8 conditions: wild type *E. coli* cells cultured at a different growth rate of 0.2, 0.5, and 0.7 per hour, and single gene knockout mutants (pgm, pgi, gapC, zwf and rpe), denoted as DataE1. To assess our method, we employed the latest updated metabolic models of *E. coli*, namely iML1515 [39]. In our comparative analysis, we also tested the previous models, iAF1260 [40] and iJO1366 [41], to showcase the benefits of using the newest metabolic model in our study.

The datasets of *S. cerevisiae* were retrieved from the study of Celton et al. [42], denoted as DataS1: four different concentrations of acetoin (0, 100, 200, and 300 mM), providing both the gene expression levels and 13C metabolic flux data under the same four conditions. The latest genome-scale metabolic model of *S. cerevisiae*, namely Yeast 8.70 [43], was utilized to evaluate our ICON-GEMs while the other *S. cerevisiae* models, namely iND750 [44] and iMM904 [45], were then used to assess the applicability of our methods.

Furthermore, to illustrate the benefits of applying co-expression networks into a genome-scale metabolic model, we also tried to create other co-expression networks from other expressions, a protein–protein interaction network, a random co-expression network, to incorporate in our ICON-GEMs. The other expression data for *E. coli* are from the studies of Zhou et al. [34] and Lacroix et al. [35], denoted as DataE2 and DataE3, respectively. For *S. cerevisiae*, we obtained the data from the studies of Rintala et al. [36] and Anders et al. [37], denoted as DataS2 and DataS3, respectively. More information about these expression datasets can be found in Additional file 2.

The random co-expression network was created by reshuffling edges to maintain the same number of edges and nodes as the original co-expression network. Protein–protein interaction networks were extracted from the String database [46] by selecting only the high confidence interactions having the confidence scores more than 900.

## Results

This section presents the results of constructing gene co-expression networks for both *E. coli* and *S. cerevisiae*, the predictive accuracy of ICON-GEMs compared to other approaches, applying various network types, subsystem flux flexibilities, and co-founding functional modules.

### Analysis of gene co-expression networks of *E. coli* and *S. cerevisiae*

The gene co-expression networks for *E. coli* and *S. cerevisiae* are established through the mapping of genes from transcriptomic data onto the genes in the genome-scale metabolic model. Measured gene expression data (DataE1 for *E. coli* and DataS1 for *S. cerevisiae*) is used to calculate Pearson correlation across various conditions. Subsequently, this Pearson correlation matrix is transformed into a binary matrix using a thresholding method. The selection of an appropriate threshold is determined by considering factors such as scale-free topology, mean connectivity, and the number of clusters.

**Fig. 2:**
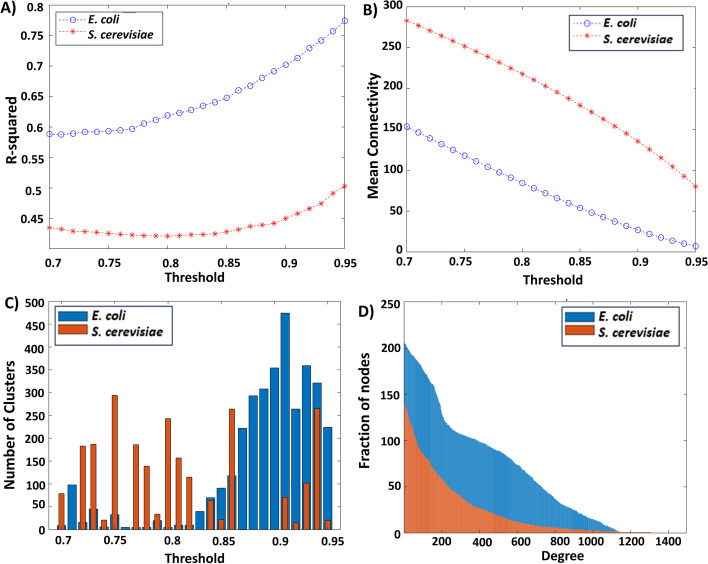
The impact of varying thresholds on the construction of gene co-expression networks for *E. coli* and *S. cerevisiae*. **A** The plot of R-squared for scale-free topology with varying thresholds. **B** The plot of mean connectivity across different thresholds. **C** The number of clusters within the gene co-expression networks constructed using various thresholds. **D** The degree distributions of the gene co-expression networks for *E. coli* with a threshold of 0.91 and for *S. cerevisiae* with a threshold of 0.94

As depicted in Fig. 2, the gene co-expression network adheres to the principles of scale-free topology at higher threshold values, contrary to the mean connectivity trend. However, constructing a network with a high threshold value results in the removal of substantial network information. Hence, a balance between achieving scale-free topology and maintaining mean connectivity is sought.

Furthermore, the determination of the number of clusters, achieved through spectrum clustering [33], plays a role in selecting the optimal threshold value. This selection process involves choosing the threshold that yields the maximum number of clusters while still adhering to the criteria of scale-free topology (with an $R^{2}$ value not less than 0.5). As mentioned in [33], weaker relationships are likely to connect functionally dissimilar segments of the network. Therefore, increasing the threshold, these segments become less interconnected, leading to an increase in the number of "nearly-disconnected" components. We choose the threshold value that maximizes the count of these components, effectively minimizing the number of edges connecting them. The chosen threshold value and the specifics of generated gene co-expression network are outlined in Table 1. Additionally, the degree distribution of each gene co-expression network is illustrated in Fig. 2(D). The degree distribution of the constructed network conforms to the principles of scale-free topology. The network exhibits a pattern where numerous nodes possess lower degrees, while a smaller number of nodes possess higher degrees.

**Table 1:** Network properties of the constructed gene co-expression networks of *E. coli* and *S. cerevisiae*

Properties	*E. coli*	*S. cerevisiae*
Selected threshold	**0.91**	**0.94**
The number of nodes	1,495	1,138
The number of edges	16,540	71,454
Average degree	22.1271	125.5782

Due to the elevated thresholds, connections conveying information about gene relationships are eliminated from the networks. The network’s structure is tailored to accommodate the information quantity and network complexity. Furthermore, the balance of connectivity alongside the data’s reduced complexity is linked to computational efficiency. The bold numbers present the selected thresholds for constructing gene co-expression networks.

### Predictive performance

ICON-GEMs’ predictive accuracy is verified by computing the uncentered Pearson product-moment correlation between in silico fluxes and corresponding 13C metabolic flux analysis for both the DC Model and AC model. We benchmark our predictive accuracy against competing methods using the same transcriptomic and fluxomic datasets. Specifically, we compare our results with E-flux [25] and E-flux2 [21], given their utilization of transcriptomic data without thresholds. Additionally, it's worth noting that the E-flux method has previously been compared with other techniques, such as GIMME [23] and iMAT [47], demonstrating superior performance in predicting exometabolomic fluxes [48] or robustness analysis [49]. The predictive accuracy assessment is carried out using transcriptomic and fluxomic data from 8 conditions within the *E. coli* model for both the DC and AC models as shown in Table 2.

**Table 2:** Predictive accuracy through the comparison of predicted fluxes and measured fluxes in eight conditions (DataE1) for both the DC and AC models

Conditions	Predictive accuracy					
	DC model			AC model		
	E-flux	E-flux2	ICON-GEMs	E-flux	E-flux2	ICON-GEMs
WT 0.2 per hour	0.8221	0.8852	**0.9443**	0.3893	**0.6293**	0.5619
$pgm$	0.8486	0.8672	**0.9639**	0.6676	0.6389	**0.6804**
$pgi$	0.8674	0.8237	**0.9107**	0.4990	**0.6129**	0.3971
$gapC$	0.6564	0.8546	**0.8828**	0.5019	**0.7012**	0.4649
$zwf$	0.8110	0.8538	**0.8731**	0.3290	**0.6499**	0.5373
$rpe$	**0.8947**	0.8885	0.8733	0.3683	0.6221	**0.7078**
WT 0.5 per hour	0.8094	0.9031	**0.9656**	0.5629	0.6615	**0.7715**
WT 0.7 per hour	0.8687	0.8699	**0.8962**	0.6711	0.6870	**0.7424**
Mean	0.8223	0.8682	**0.9206**	0.1225	**0.6504**	0.6079
Standard Deviation	0.0688	0.0231	0.0333	0.1555	0.0292	0.1284

The bold numbers indicate the highest predictive accuracy values for each condition

Eight distinct conditions encompass wild-type *E. coli* growth rates at 0.2 (as reference (RF)), 0.5 (WT0.5), and 0.7 (WT0.7) per hour as well as specific gene deletions (of genes pgm, pgi, gapc, zwf, and rpe)

Notably, our method consistently achieves predictive accuracy exceeding 0.87 in all conditions for the DC model. The average performance for our method in this model is 0.9206, indicating high accuracy. In the AC model, the average performance is 0.6079. While this value may not be exceptionally high, an accuracy around 0.6 denotes a moderate positive correlation between the predicted fluxes and measured fluxes.

The performance of ICON-GEMs in predicting fluxes for four conditions within Yeast 8.70, a multi-compartment genome-scale metabolic model of *S. cerevisiae*, is shown in Table 3 for both the DC and AC models. The average performance is 0.9689 (standard deviation: 0.0357) for the DC model and 0.6272 (standard deviation: 0.2715) for AC model.

**Table 3:** Predictive accuracy through the comparison of predicted fluxes and measured fluxes in four conditions (DataS1) for both the DC and AC model

Conditions	Predictive accuracy					
	DC model			AC model		
	E-flux	E-flux2	ICON-GEMs	E-flux	E-flux2	ICON-GEMs
0 mM	0.9266	**0.9643**	0.9074	0.4093	0.4509	**0.5666**
100 mM	0.9508	0.9825	**0.9850**	0.4219	**0.6141**	0.5857
200 mM	0.5979	0.8807	**0.9872**	0.4123	0.4554	**0.9417**
300 mM	0.9763	0.9763	**0.9959**	0.4231	**0.4244**	0.4149
Mean	0.8629	0.9522	**0.9689**	0.4167	0.4862	**0.6272**
Standard Deviation	0.1539	0.0421	0.0357	0.0059	0.0748	0.2715

The bold numbers indicate the highest predictive accuracy values for each condition

These conditions correspond to different concentrations of acetoin in *S. cerevisiae*; specifically, at 0 mM (as reference (RF)), 100 mM, 200 mM, and 300 mM

Comparatively, the performance of our method in DC models surpass those of E-flux and E-flux2 across all conditions. For AC models, our method consistently outperforms E-flux and, in some conditions, E-flux2. On average, our method performs better than both E-flux and E-flux2 in both model types. From the results, we deduce that our method excels in the DC models compared to E-flux and E-flux2 methods. In the AC models, our method outperforms E-flux but lags behind E-flux2. Our approach is most suitable for modeling scenarios with known carbon sources.

Furthermore, we visualize the distribution comparison between predicted and measured fluxes in *E. coli* and *S. cerevisiae*. Given the absence of units for predicted fluxes, we normalize their magnitudes to enable direct comparison with measured fluxes. The visualization focuses on wildtype at a growth rate of 0.5 per hour for *E. coli* and an acetoin concentration of 200 mM for *S. cerevisiae*, which yielded the highest performance.

**Fig. 3:**
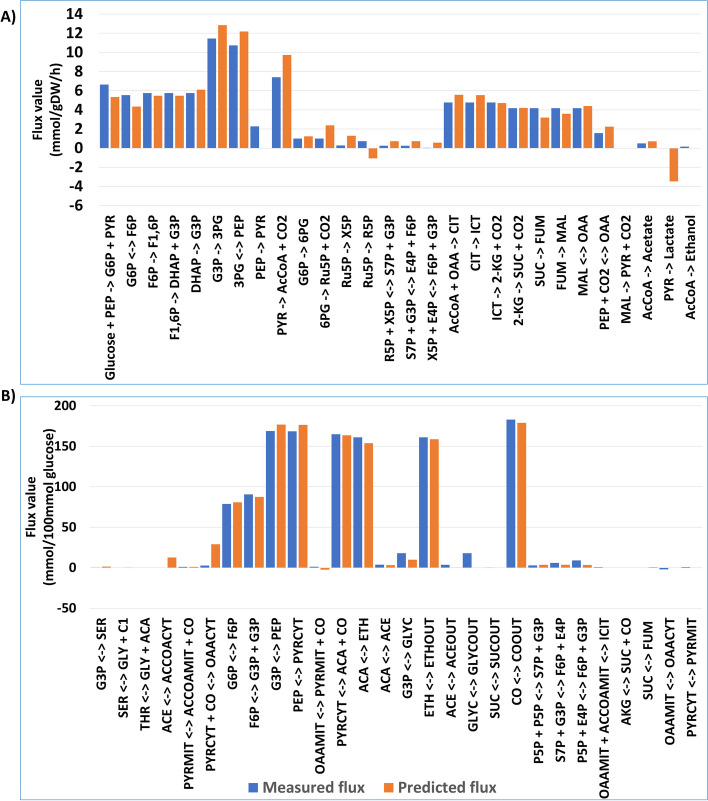
The comparison entails a visual examination of predicted fluxes against measured fluxes. This comparison is conducted within the context of wildtype conditions at a growth rate of 0.5 per hour for *E. coli* **A**, and in presence of acetoin at concentration of acetoin 300 mM for *S. cerevisiae* **B** in the DC models

**Fig. 4:**
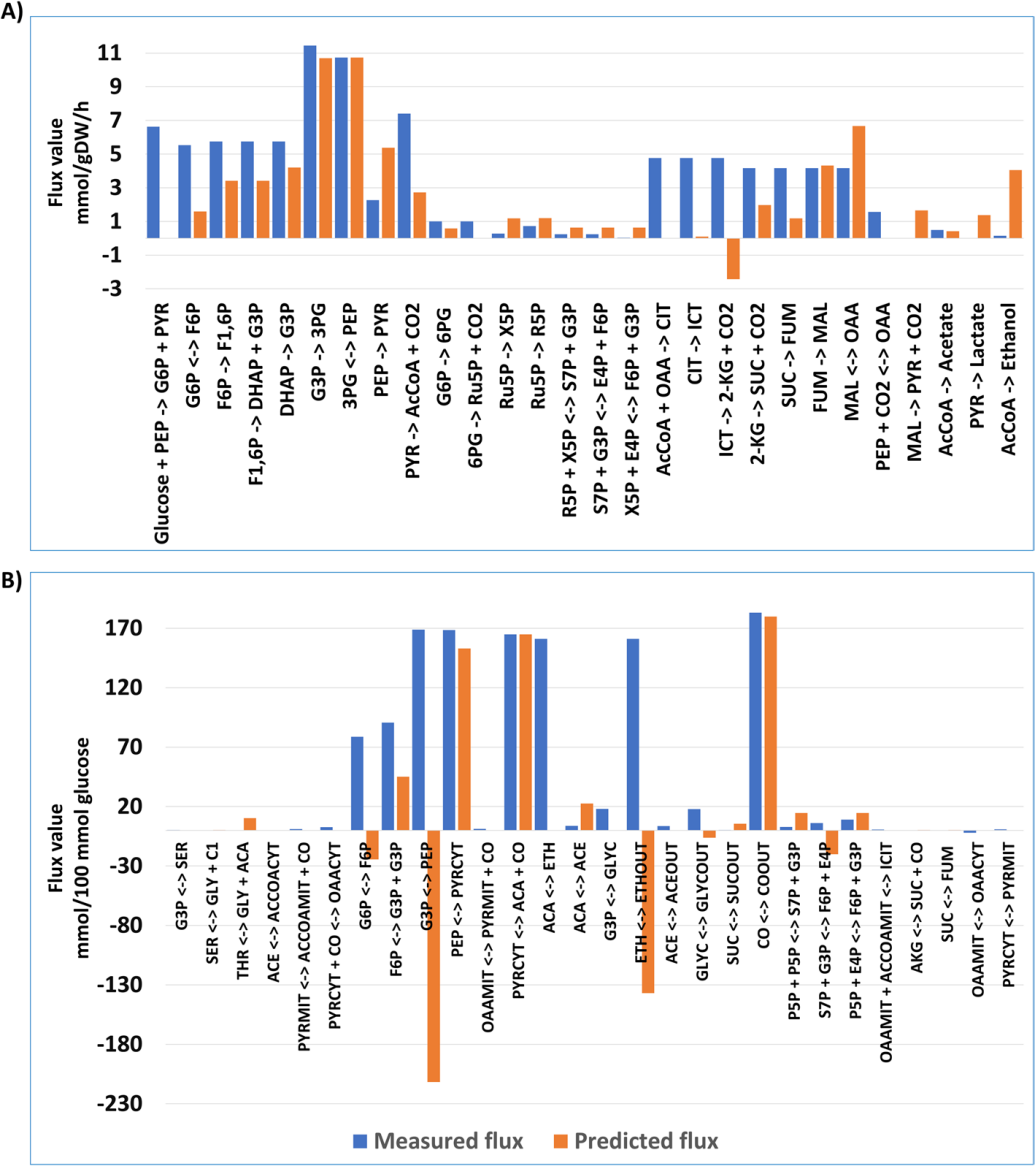
The comparison entails a visual examination of predicted fluxes against measured fluxes. This comparison is conducted within the context of wildtype conditions at a growth rate of 0.5 per hour for *E. coli* **A**, and in presence of acetoin at concentration of acetoin 300 mM for *S. cerevisiae* **B** in the AC models

Figure 3 visually depicts the distribution comparison between predicted and measured fluxes for *E. coli* and *S. cerevisiae*. Given the absence of units for predicted fluxes, their magnitudes are normalized for direct comparison with measured fluxes. The comparison is particularly emphasized in the DC models, yielding consistency between predicted and measured fluxes, as evidenced by Fig. 3(A) and (B) for the DC models in *E. coli* and *S. cerevisiae*, respectively. However, AC models display inconsistencies between predicted and measured fluxes. The corresponding visualizations for the AC models are available in Fig. 4 (A) and (B), for *E. coli* and *S. cerevisiae*, respectively. The *x*-axis represents measured fluxes while the *y*-axis represents predicted flux values.

### Capability of flux predictions by ICON-GEMs on previously established models

Before applying our method to a wide range of living organisms, it is important to make sure that our technique works well even with models that are not fully complete. Right now, there are still ongoing efforts to create comprehensive models of the metabolic processes in various organisms. To thoroughly test our method, we use the same kind of data on gene activity and the flow of substances through the cells for organisms whose metabolic models are already established. Specifically, we use our method on the metabolic models of *E. coli* (iAF1260 [40] and iJO1366 [41]) and *S. cerevisiae* (iND750 [44] and iMM904 [45]).

**Fig. 5:**
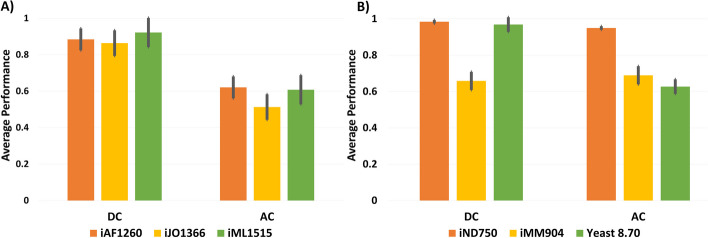
Comparison of average performances in ICON-GEMs for previous and recent models of *E. coli* (**A**) model *S. cerevisiae* (**B**)

The results of this testing on the existing models are shown in Fig. 5(A) and (B). The horizontal axis represents two types of metabolic models: DC and AC models. Figure 5(A) pertains to *E. coli*, while Fig. 5(B) focuses on *S. cerevisiae*. The models are grouped into two categories based on whether they are DC or AC models. With in each group, three bars are depicted, corresponding to the oldest, middle, and newest models, which are represented by orange, yellow, and green bars, respectively. On the vertical axis, we display the average similarity between predicted and actual substance flow rates. The lines on top of the bars show how much the values vary.

Regarding *E. coli*, as shown in Fig. 5A, all DC models produced average performance exceeding 0.8, indicating high levels of accuracy. Similarly, all AC models yielded average performance surpassing 0.5. Notably, the newest model (iML1515) demonstrated the highest performance in both the DC and AC models. Turning to *S. cerevisiae*, illustrated in Fig. 5B, the earliest model iND750 exhibited the highest average performance in both DC and AC models. The newest model also displayed a notably high average performance. In contrast, the performance for using iMM904 was inferior to that of iND750 and Yeast 8.70 in both the DC and AC models.

Considering our method's capacity to predict flux in incomplete models, it demonstrates effective performance in predicting metabolic fluxes within such contexts. While there may be instances of less accurate results, a predictive performance greater than 0.5 indicates a moderate level of correlation between predicted fluxes and measure fluxes. As shown in the results above, the DC model consistently outperforms the AC model. Therefore, in the subsequent sections, we refine our study by narrowing our focus to the DC model exclusively, and no longer consider both the DC and AC models.

### Flexibility of reaction fluxes in subsystems

**Fig. 6:**
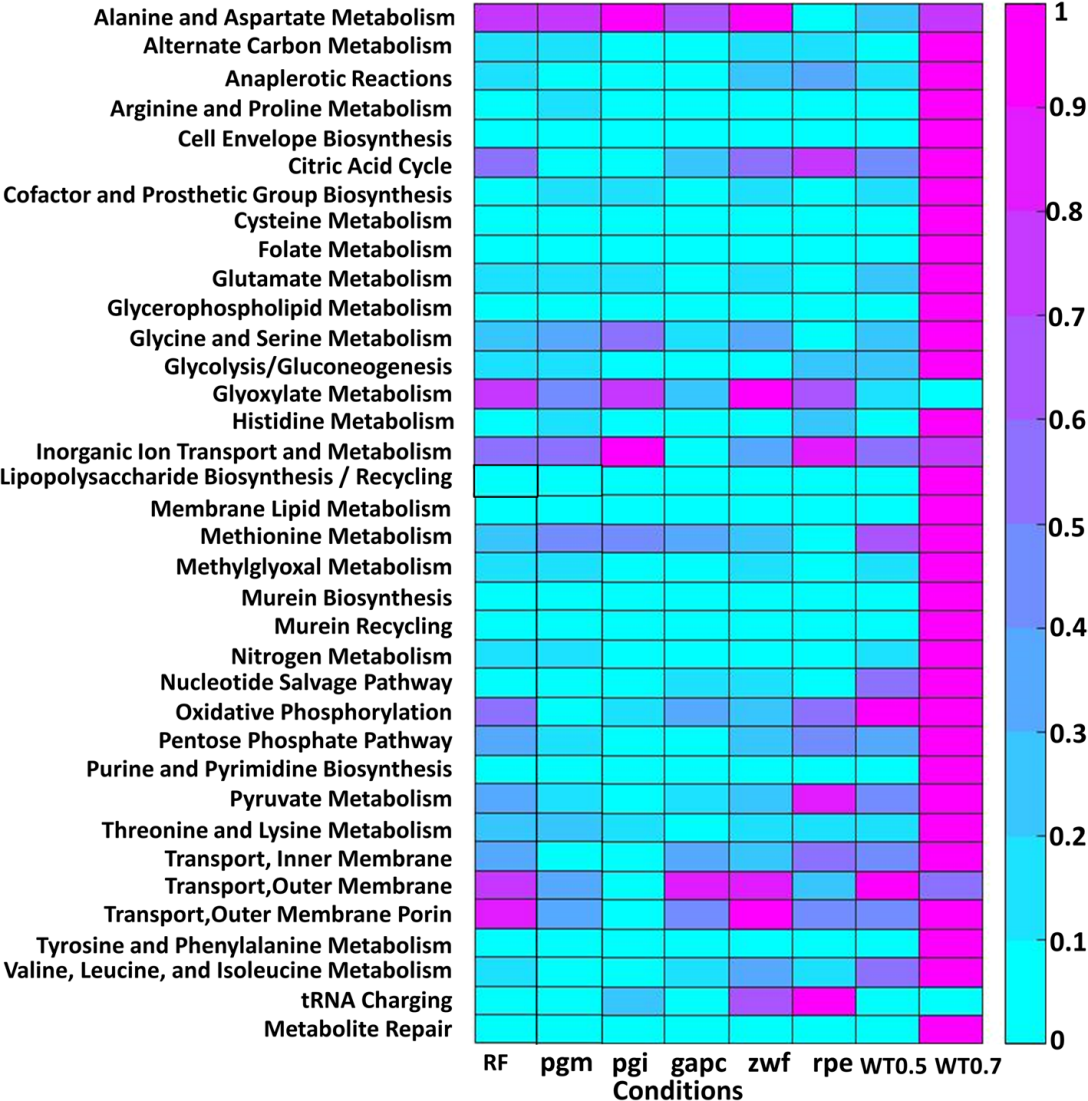
The heatmap shows average of flexibility of reaction fluxes within various subsystems under eight distinct conditions. These conditions encompass wild-type *E. coli* growth rates at 0.2 (as reference (RF)), 0.5 (WT0.5), and 0.7 (WT0.7) per hour as well as specific gene deletions (of genes pgm, pgi, gapc, zwf, and rpe)

**Fig. 7:**
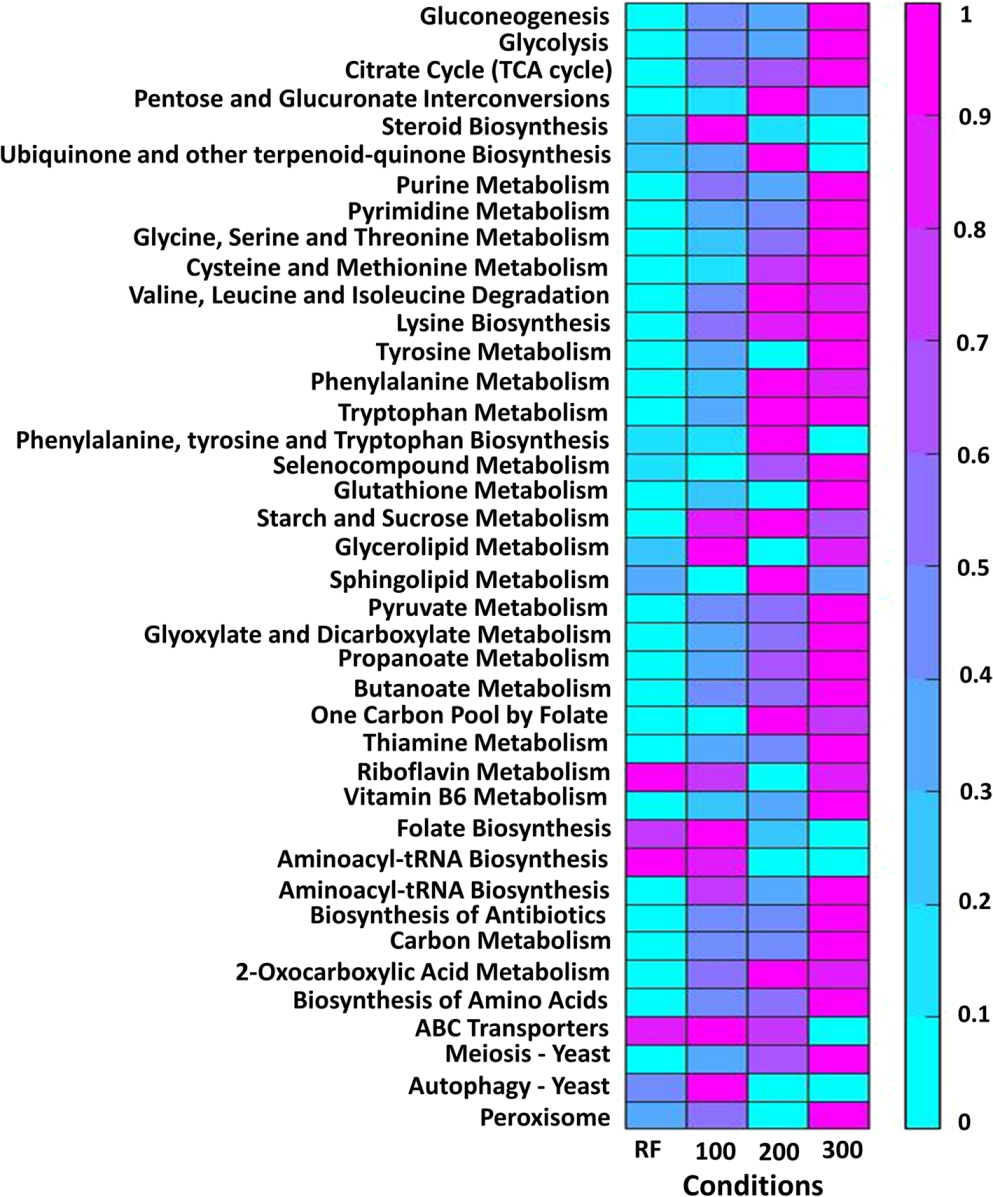
The heatmap shows average of flexibility of reaction within various subsystems under four distinct conditions. These conditions correspond to different concentrations of acetoin in *S. cerevisiae*; specifically, at 0 mM (as reference (RF)), 100 mM, 200 mM, and 300 mM

The integration of condition-specific experimental data into the metabolic system’s constraints leads to a reduction in the solution space and range of reaction fluxes. The flexibility of a reaction is the difference between maximum flux and minimum flux of that reaction as mentioned in the method section. The outcomes of detailing the flexibility of reaction fluxes within each subsystem of *E. coli* and *S. cerevisiae* can be found in Figs. 6 and 7. These figures depict heatmaps showcasing the average difference between potential maximum and minimum reaction fluxes for each reaction within a subsystem, normalized to a range of [0,1]. Higher values indicate significant variability in flux through a subsystem while still maintaining a defined value of the objective function in the first stage programming. The findings reveal that the flux flexibility remains consistent with the model that does not consider a gene co-expression network. This suggests that our model can adapt to various scenarios of flux availability and uphold the flux overflows seen in the original model. In essence, incorporating co-expression information, our model offers more precise relative flux values compared to using a single condition.

Our method establishes flexible fluxes guided by gene expression values. Despite multiple solutions yielding the same optimal objective value, the flux range remains constrained by gene expression data, ensuring quality solutions. The flexibility level is particularly pronounced in wildtype conditions at a growth rate of 0.7 per hour for *E. coli* and at an acetoin concentration of 300 mM for *S. cerevisiae*.

### Effect of various biological networks incorporating with genome-scale metabolic models

Since the formulated quadratic programming primarily revolves around an objective function linked to the presence of gene co-occurrence or co-expression within a network, it serves to highlight the advantages of employing a co-expression network over alternative biological networks, such as protein–protein interactions (PPIs). In order to ascertain the efficacy of integrating a gene co-expression network into a metabolic model for more accurate flux distribution outcomes, we accomplish this by substituting a gene co-expression network with various biological networks, including gene co-expression networks from other datasets (DataE1, DataE2, and DataE3) and PPI networks while the conditions of interest remained the same. Additionally, we explore the adaptability of the gene co-expression network by subjecting it to random modifications of edge connections at varying percentages (random networks). The results of integrating different biological networks with the genome-scale metabolic model is presented in Table 4 for *E. coli* and Table 5 for *S. cerevisiae*. The random network results were derived from 10 repetitions. The predictions made when utilizing PPI were based on the expression data DataE1 for *E. coli* and DataS1 for *S. cerevisiae*.

**Table 4:** Predictive accuracy when applying various biological networks incorporating with the genome-scale metabolic model in *E. coli*

Conditions	DataE1 (Own)	DataE2 (Microarray)	DataE3 (RNAseq)	PPI	Random (% of edges)			Random network
					25	50	75	
WT 0.2/h	0.9443	0.9011	0.8852	0.8881	0.9401	0.9438	0.8869	0.8895
pgm	0.9639	0.9313	0.8064	0.7516	0.9556	0.9115	0.9215	0.9193
pgi	0.9107	0.8979	0.8888	0.8894	0.9106	0.9007	0.9105	0.9133
gapC	0.9287	0.8901	0.8741	0.8741	0.9215	0.8753	0.8753	0.8796
zwf	0.8828	0.8412	0.8252	0.8139	0.8722	0.8275	0.8319	0.8311
rpe	0.8731	0.8243	0.7918	0.8286	0.8631	0.8071	0.8071	0.8189
WT 0.5/h	0.9656	0.8660	0.9001	0.8660	0.9588	0.9005	0.9007	0.9025
WT 0.7/h	0.8962	0.8357	0.8643	0.8357	0.9088	0.8410	0.8684	0.8662
Mean	0.9206	0.8434	0.8545	0.8434	0.9164	0.8759	0.8753	0.8775
Standard deviation	0.0333	0.0354	0.0383	0.0435	0.0332	0.0439	0.0366	0.0345

Eight distinct conditions encompass wild-type *E. coli* growth rates at 0.2 (as reference (RF)), 0.5 (WT0.5), and 0.7 (WT0.7) per hour as well as specific gene deletions (of genes pgm, pgi, gapc, zwf, and rpe)

**Table 5:** Predictive accuracy when applying various biological networks incorporating with the genome-scale metabolic model in *S. cerevisiae*

Acetoin	DataS1 (Own)	DataS2 (Microarray)	DataS3 (RNAseq)	PPI	Random (% of edges)			Random network
					**25**	**50**	**75**	
0 mM	0.9074	0.8919	0.8922	0.9936	0.8909	0.8944	0.8248	0.8570
100 mM	0.9850	0.8961	0.9006	0.8948	0.8311	0.8115	0.8133	0.8047
200 mM	0.9872	0.8966	0.9079	0.8999	0.9010	0.8959	0.8842	0.8245
300 mM	0.9959	0.9019	0.9022	0.8998	0.8381	0.7941	0.8016	0.7945
Mean	0.9688	0.8966	0.9007	0.9221	0.8653	0.8490	0.8310	0.8201
Standard deviation	0.0357	0.0035	0.0055	0.0413	0.0310	0.0466	0.0318	0.0309

These conditions correspond to different concentrations of acetoin in *S. cerevisiae*; specifically, at 0 mM (as reference (RF)), 100 mM, 200 mM, and 300 mM

The results showcasing the integration of gene co-expression networks from alternative datasets (DataE2 and DataE3 for *E. coli*, DataS2 and DataS3 for *S. cerevisiae*), in Tables 4 and 5, provide some interesting biological insights. In the case of *E. coli*, when integrating gene co-expression networks based on DataE2, DataE3, DataS2, DataS3 and PPI networks, the predictive accuracy is weaker compared to the integration of the original gene co-expression network. It is worth mentioning that while DataE2, DataS2 are from microarray data, DataE3 and DataS3 stem from RNA-seq technology.

Furthermore, we subject the gene co-expression network to random edge perturbations at levels of 25%, 50%, 75%, and 100% (random networks) to assess the resilience of ICON-GEMs. The outcomes reveal that at 25%, 50%, and 75%, the flux correlation experiences slight reductions compared to the gene co-expression network due to the relative stability of hub genes. However, at 100%, the altered hub genes lead to a drop in flux correlation compared to the baseline network integration for both *E. coli* and yeast models.

### Impact of various conditions of datasets for gene co-expression network construction

**Fig. 8:**
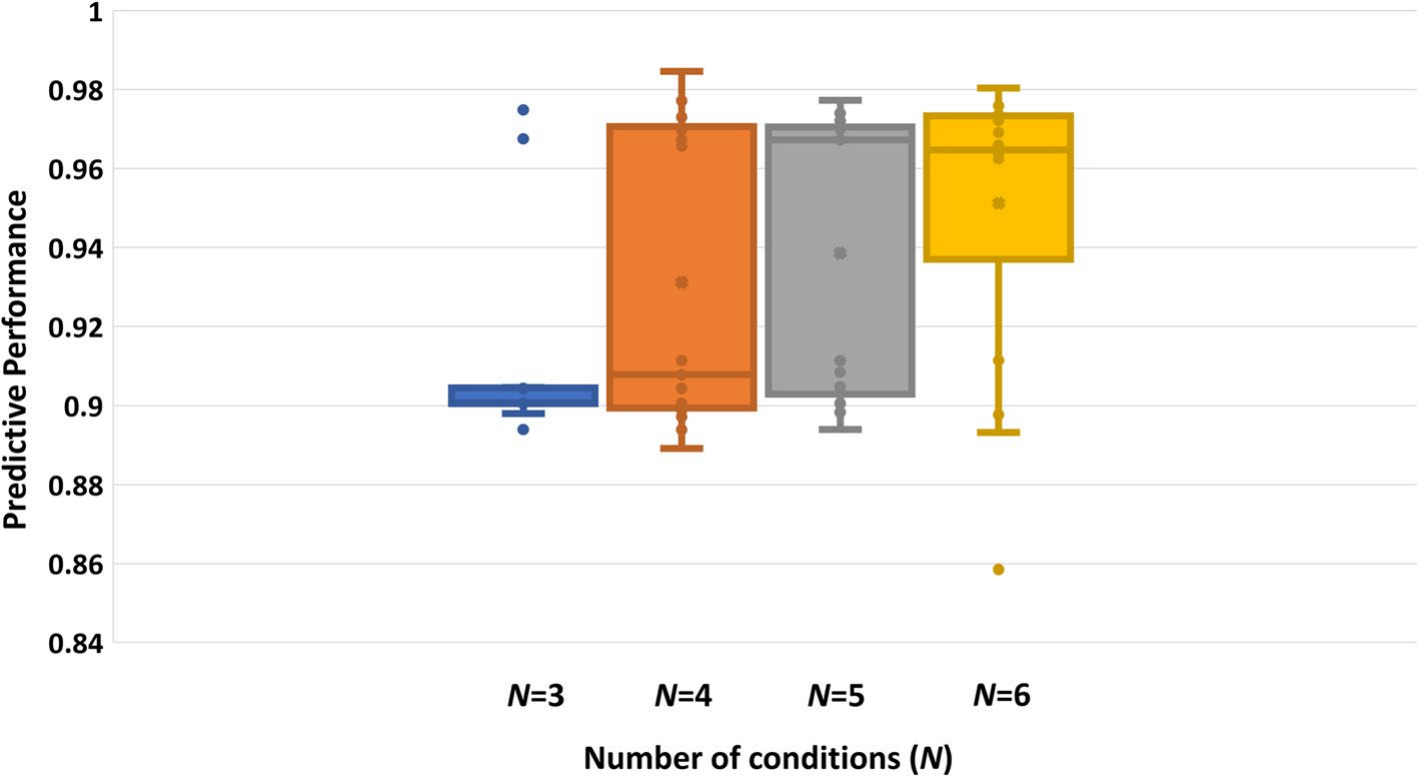
The predictive performance for WT at 0.5 per hour varies with the number of conditions (*N*) used in the gene co-expression construction model

Constructing a gene co-expression matrix is crucial for the method’s performance. To extract gene co-expression relationships, a substantial number of transcriptome datasets from various conditions is necessary. In contrast to tools like GIMME or E-flux, which only require a single-condition transcriptome, ICON-GEM necessitates data from multiple conditions. Performance may vary when the combination or quantity of datasets changes, even if the specific condition of interest remains constant. We assessed the robustness of ICON-GEM by creating gene co-expression networks with various dataset combinations, as shown in Fig. 8 and elaborated in Additional file 3. We conducted this evaluation by integrating each obtained network into the metabolic network under wildtype growth conditions at a rate of 0.5 per hour. The gene co-expression networks were constructed via various numbers of conditions, denoted as *N*. We observed that when *N* = 2, all genes in the co-expression network were highly correlated or connected because there were only two data points for calculating Pearson correlation. The number of conditions significantly influences gene relationships. Thus, with *N* = 2, extracting meaningful information from only two conditions becomes challenging.

Based on Fig. 8, when *N* is greater than 2, the performance consistently exceeded 0.88. This suggests a strong relationship between the gene co-expression network and metabolic network integration. Furthermore, as the number of conditions increases, the average performance, measured by the uncentered Pearson product-moment correlation coefficient between predicted and measured fluxes, also increases. These results highlight the robustness and reliability of the integrated gene co-expression network; especially, when utilizing a greater number of conditions.

### Concordance between gene co-expression modules and reaction fluxes

Understanding the intricate interplay between gene expression patterns and metabolic activity is crucial for unraveling the regulatory mechanisms within biological systems. In this study, we delve into the relationship between gene co-expression modules and reaction fluxes in the context of the *E. coli* and Yeast organisms. The initial step involved the application of TOM (Topological Overlap Measure) similarity [50, 51] and hierarchical clustering techniques to the gene co-expression network. By employing TOM similarity, we assessed the topological overlap of gene expression profiles, allowing us to identify genes with similar expression patterns. Hierarchical clustering then facilitated the grouping of these genes into distinct modules, each characterized by a specific set of co-expressed genes.

This clustering process yielded nine discrete modules for *E. coli* data and six modules for the Yeast data. The list of all modules and their gene members of *E. coli* and *S. cerevisiae* can be found in Additional file 4. In each module, we utilized the DAVID tool (Database for Annotation, Visualization, and Integrated Discovery) to identify pertinent terms from Gene Ontology (GO) and Kyoto Encyclopedia of Genes and Genomes (KEGG) pathways. This approach allowed us to pinpoint the most statistically significant GO and KEGG terms within each module. Detailed GO and KEGG enrichment results can be found in Additional file 5. These tables furnish insights into the annotated pathways and biological functions linked to the respective gene modules in each organism.

Subsequently, we sought to examine the concordance between these gene co-expression modules and the reaction fluxes occurring within the metabolic network via the predictions from ICON-GEMs and E-flux method. Reaction fluxes represent the flow of metabolites through metabolic pathways, reflecting the actual functional activity of the network. By analyzing the correspondence between gene modules and reaction fluxes, we gained insights into the alignment of transcriptional patterns with metabolic behavior.

**Fig. 9:**
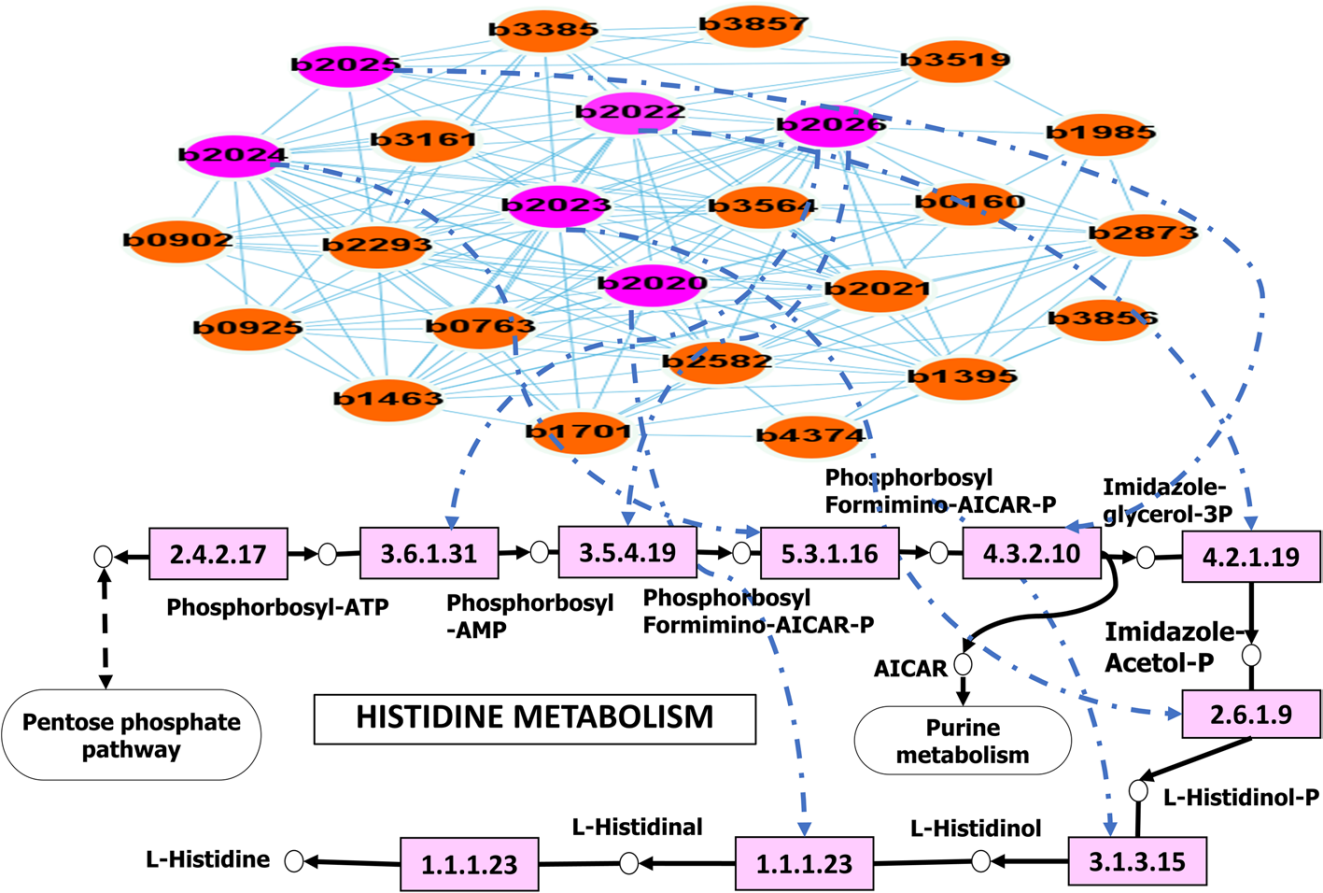
Histidine metabolism in module ME7 of *E. coli* to represent the control of the gene co-expression network onto a metabolic process. The pink nodes are genes associated with enzymes in this pathway. The other nodes are genes connected with the pink nodes in the co-expression network

To better understand how the ICON-GEMs incorporate the gene co-expression network onto the metabolic pathway, we illustrate the histidine metabolism and their gene co-expression network, which is found to be enriched in module ME7 for *E. coli*, as shown in Fig. 9. The red and pink nodes in the figure represent genes within module ME7. The pink nodes correspond to genes associated with enzymes in the histidine metabolism pathway. The others are genes that co-related with the pink nodes in the co-expression network. Totally, there are ten enzymes involved in this pathway and six genes (b2020, b2022, b2023, b2024, b2025, and b2026) found to control the process of this pathway. According to the defined quadratic programming model in ICON-GEMs, the boundaries of these reaction fluxes were not only controlled by the direct transcriptomic expression levels but also by the collaborative gene partners found in co-expression network analysis. Therefore, the regulation for this histidine pathway as shown in Fig. 9 involved with the other genes (red nodes in the figure) in cooperating its own process and some other involved processes based on the co-expression network as well. Our findings reveal intriguing relationships between gene co-expression modules and reaction fluxes, highlighting instances of coordinated regulation where groups of co-expressed genes correspond to specific metabolic pathways. This concordance underscores the significance of transcriptional regulation in influencing metabolic outcomes and suggests potential nodes of regulatory control within the metabolic system.

**Fig. 10:**
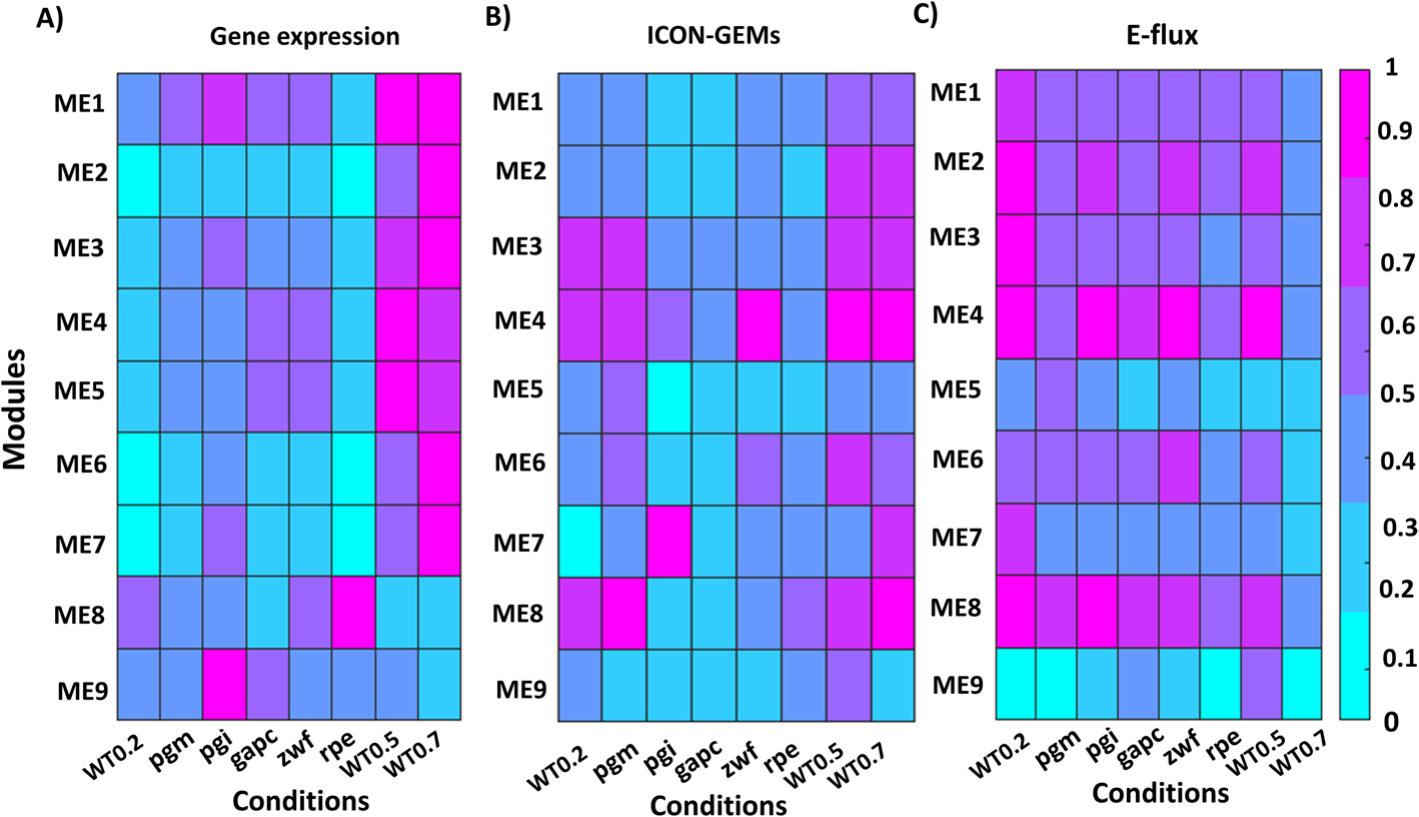
The heatmap shows the average of **A** gene expression and reaction fluxes calculated by **B** ICON-GEMs and **C** E-flux method of *E. coli* in each module. Eight distinct conditions encompass wild-type *E. coli* growth rates at 0.2 (as reference (RF)), 0.5 (WT0.5), and 0.7 (WT0.7) per hour as well as specific gene deletions (of genes pgm, pgi, gapc, zwf, and rpe)

**Fig. 11:**
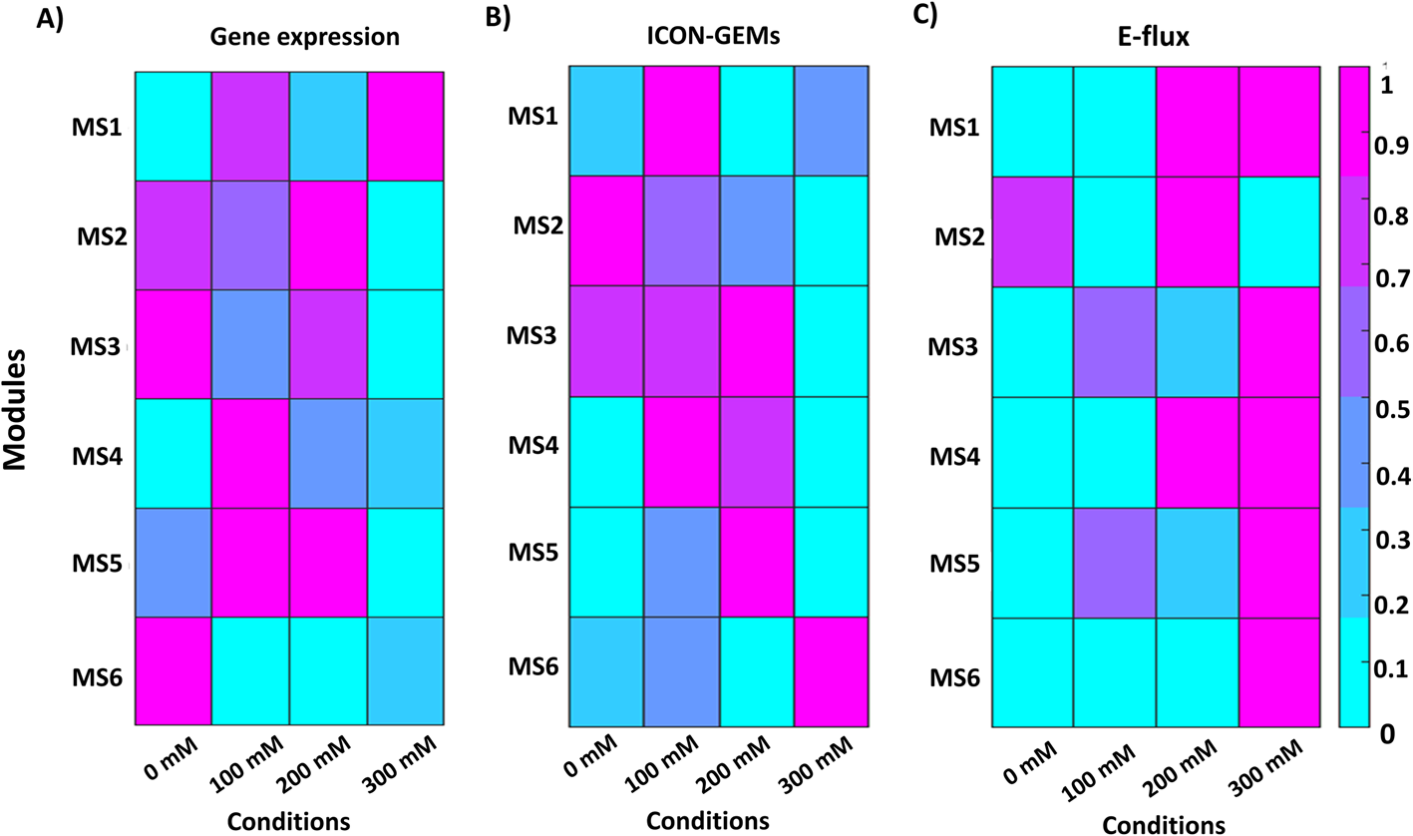
The heatmap shows the average of **A** gene expression and reaction fluxes calculated by **B** ICON-GEMs and **C** E-flux method of *S. cerevisiae* in each module. These conditions correspond to different concentrations of acetoin in *S. cerevisiae*; specifically, at 0 mM (as reference (RF)), 100 mM, 200 mM, and 300 mM

Furthermore, we executed a comparative examination of gene expression data and reaction flux within each module, as illustrated in Fig. 10 for *E. coli* and Fig. 11 for *S. cerevisiae*. This visual representation displays a heatmap depicting the normalized averages of gene expression levels and reaction fluxes from our ICON-GEMs and E-flux technique, across the diverse modules. As demonstrated by Figs. 10 and 11, a noticeable alignment emerges between the average metabolic flux and gene expression data within each module. For *E. coli* and *S. cerevisiae* model, the heat map of clustering-derived average flux and gene expression values exhibit consistent concordance to our ICON-GEMs results rather than the outcomes of the E-flux method. The incorporation of the gene co-expression network into the metabolic framework significantly bolsters the coherence of average metabolic flux with the corresponding gene expression data within each module. In summary, our investigation sheds light on the interconnectedness of gene expression and metabolic function by examining the congruence between gene co-expression modules and reaction fluxes. This integrative approach not only advances our understanding of the regulatory landscape in *E. coli* and *S. cerevisiae* but also provides a framework for dissecting similar relationships in other biological contexts.

## Discussion

The ICON-GEMs present an inclusive strategy to merge co-expression networks with metabolic models, offering a means to uncover functional associations between genes and metabolic pathways. This approach employs quadratic programming with an objective function involving the summation of products of reaction flux pairs corresponding to gene pairs exhibiting high correlations, as indicated by the gene co-expression network.

We evaluated our method using gene expression data and GEMs of *E. coli* and *S. cerevisiae*. The results showed that the flux distributions obtained through the quadratic programming approach closely align with the experimental outcomes from 13C metabolic flux analysis under both the DC and AC models. Additionally, our approach exhibited a significant ability to predict flux within earlier GEM models, demonstrating remarkable accuracy in most cases and moderate performance for models with less completion. Moreover, the reaction flexibility for each subsystem in our approach, calculated through flux variability analysis, is consistent with the original results of the model without the gene co-expression network. Finally, our method of adding the gene co-expression clearly demonstrates the natural consistence between gene module clusters and reaction fluxes better than the method of using a single value of gene expression level to regulate fluxes. This directly highlights the benefits of employing a gene co-expression network to regulate metabolic processes.

The main factor in constructing an accurate gene co-expression network involves preparing gene expression profiles and calculating correlation coefficient between gene pairs. The collection of data on gene expression should be driven by the conditions that are of particular interest for study, and these conditions should be selected to effectively reveal the relationships between the genes under investigation. We applied our approach to *E. coli* and *S. cerevisiae*, well-studied organisms with relatively accurate models due to their extensive research history. Challenges still persist when applying ICON-GEMs to other organisms, especially those that have not been extensively studied in the context of metabolism. In such cases, there may indeed be a need for additional research to enhance our understanding of their metabolic processes. To calculate the correlation coefficients, we applied Pearson for capturing linear relationships between any two genes. This calculation cannot capture non-linear or more complex relationships. In principle to calculate linear or non-linear correlations, we require more than two data points. Thus, the application of this coefficient for constructing a gene co-expression network requires more than two conditions. Therefore, it is important to note that, unlike methods such as E-flux and GIMME, which rely on a single condition to regulate metabolic flows, our approach, ICON-GEMs, leverages multiple conditions to extract gene relationships. Consequently, to construct a gene co-expression network that better predicts fluxes, ICON-GEMs requires the utilization of more than two conditions. Moreover, with an increase in the number of conditions, there is a corresponding improvement in the average performance.

The transformation of correlation coefficients into a binary adjacency matrix serves to establish the co-expression network. This binary representation simplifies the network structure by focusing on the presence or absence of a relationship between genes. The approach of employing a threshold to determine network edges introduces a critical decision point. The balance between achieving scale-free topology and maintaining mean connectivity determines the network's complexity and information content. This trade-off highlights the intricate nature of biological networks, where both connectivity patterns and global network properties play roles in understanding cellular processes.

ICON-GEMs typically involve sets of gene pairs within a gene network. Therefore, any network type can be used to relate gene relationships to a metabolic network, as demonstrated in the results. It turns out that the gene co-expression network utilizing linear correlation in this study exhibits better performance than the other networks. It remains open to explore the use of non-linear correlations for gene co-expression networks with gene expression data in the future. We also show that the superiority of gene co-expression network over alternative networks like gene co-expression networks with the other data sources, protein–protein interactions (PPIs) in capturing gene relationships, and random networks with the same number of genes and connections, on metabolic flux predictions. Interestingly, ICON-GEMs detected an important result when using the co-expression network generated from related experiments slightly better than the original expression data set. It might be because using the original expression data to build up the co-expression network would provide an overfitting problem to lose some crucial complex relationships among genes. Using co-expression from similar or related experiments would provide more flexibility to detect broader co-partners in metabolic processes. It is a hint for the importance of the era of integrating multi-data set from diverse experimental techniques.

Finally, it is important to acknowledge that our ICON-GEMs involves nonconvex quadratic programming, presenting challenges in terms of processing speed due to its NP-hard nature. However, with advancements in computational technology and storage capacity, it is still feasible to calculate flux solutions for a genome-scale metabolic model with a gene co-expression network in a reasonable timeframe. Nonetheless, ICON-GEMs provide a novel perspective on the interdependencies of reaction fluxes, shedding light on unexplored aspects of cellular metabolism.

## Conclusion

 Analyzing flux distribution at the condition level holds significant importance across various applications. Conventional enhanced Flux Balance Analysis (FBA) methods typically rely solely on gene expression values, overlooking the intricate gene correlations. In contrast, ICON-GEMs introduces a pioneering computational approach that enables the quantification of flux distribution while concurrently integrating gene co-expression networks. The flux distribution generated by ICON-GEMs closely aligns with experimental findings. Remarkably, ICON-GEMs outperform existing prediction methods, yielding results of heightened accuracy. Notably, this approach unveils insightful metabolic pathways linked to the provided transcriptomic data. Furthermore, ICON-GEMs boasts versatility, as it can be effectively applied to diverse transcriptomic data and various organisms.

### Supplementary Information

**Additional file 1.** The Formulation Details of ICON-GEMs.

**Additional file 2.** Details of Transcriptomic, Fluxomic, and Metabolic Model Datasets.

**Additional file 3.** The properties of the gene co-expression network across different numbers of conditions.

**Additional file 4.** The list of all modules and their gene members in *E. coli* and *S. cerevisiae*.

**Additional file 5.** The detailed results of Gene Ontology (GO) and KEGG pathway enrichment analysis.

## Data Availability

All data generated or analyzed in this study can be found in the supplementary information files and/or at GitHub site at https://github.com/ThummaratPaklao/ICOM-GEMs.git
