# Supplementary material for: ICON-GEMs: integration of co-expression network in genome-scale metabolic models, shedding light through systems biology
Source: BMC Bioinformatics. 2023 Dec 21;24:492. doi: 10.1186/s12859-023-05599-0 (PMC10740312; doi:10.1186/s12859-023-05599-0)
Supplement: Supplementary file 1 — Additional file 1. The Formulation Details of ICON-GEMs. [file 12859_2023_5599_MOESM1_ESM.docx]

**Additional File 1: Formulation of ICON-GEMs**

**S1: Flux balance Analysis**

Flux balance analysis (FBA) is as a linear programming or computational approach to determine the steady-state flow of metabolites through a metabolic network. It is a well-established method in the field of metabolic systems.

Let $v$ be the vector of reaction flux and $S$ be the stoichiometric matrix. Under the assumption of Flux Balance Analysis (FBA), each internal metabolite is consumed at the same rate at which it is produced. This assumption implies that the system of equations $Sv$ must equal zero.

Hence, linear programming or optimization techniques are employed to identify and analyze the optimal solution within a constrained space. This encompasses determining the concentration of metabolites at the steady-state and establishing the range of reaction flux that can be used to set the upper ($U$) and lower ($L$) bounds of $v$. The objective function is used to quantify the contribution of each component towards achieving the desired flux.

**S2: Construction of Template Metabolic models**

ICON-GEMs requires the gene expression data for incorporating into the genome-scale metabolic model. Given the inherent disconnect between the units used to measure metabolic reaction flux and gene expression data, the creation of a template metabolic model is a solution. This template model preserves stoichiometric and reversibility information from the original genome-scale metabolic model while excluding specific flux rate data. The template model is constructed by setting the bounds of reaction flux to either zero or to a predefined large value denoted as $T$.

Suppose that there are m metabolites and $n$ reactions in a metabolic network. Let $\hat{L}$ and $\hat{U}$ be the new lower and upper bound of flux of reaction in template metabolic model, respectively:

$\hat{L}_{j}=\left\{ \begin{aligned} 0 if L_{j}\geq0 \\ -T if L_{j}<0 \end{aligned} \right.$ and $\hat{U}_{j}=\left\{ \begin{aligned} T if U_{j}>0 \\ 0 if U_{j} \leq0 \end{aligned} \right.$ for all $j=1,2,3,...,n$.

In some situations, the used carbon sources in the cell are unknown. Thus, this template metabolic model is constructed in two different cases depending on the information of carbon source.

The first template metabolic model, known as the DC (Determined Carbon Source) model, sets the lower bound of the known carbon source reaction fluxes to a negative value of the largest number. The known carbon source for DC model is glucose since the experimental data that is used to demonstrate in this study were measured by feeding glucose into the system.

**DC Model**

**-T**

The second model is called AC (all possible carbon source) model which defines a negative value of the largest number to the lower bound of all carbon source reaction flux in the metabolic model.

**-T**

**AC Model**

**-T**

**-T**

**-T**

**-T**

**S3: Irreversible model**

In a metabolic network, the sign of a reaction flux indicates the direction of the reaction. Consequently, reversible fluxes may exhibit negative values. To simplify calculations, reversible reactions are often transformed into two separate irreversible reactions. Let $v^{rev}$ be vector of reversible reaction flux.

Assume that there are $p$ reversible reactions within metabolic network. Let $v_{j}^{a}$ represent the forward and $v_{j}^{b}$ represent the backward reaction flux for reaction $j$. Then

$v_{j}^{rev}=v_{j}^{a}-v_{j}^{b}$ (2)

Where $v_{j}^{a},v_{j}^{b}\geq0$ for all $j=1,2,3,\ldots,p$.

**S4: Mapping of gene expression data into metabolic models**


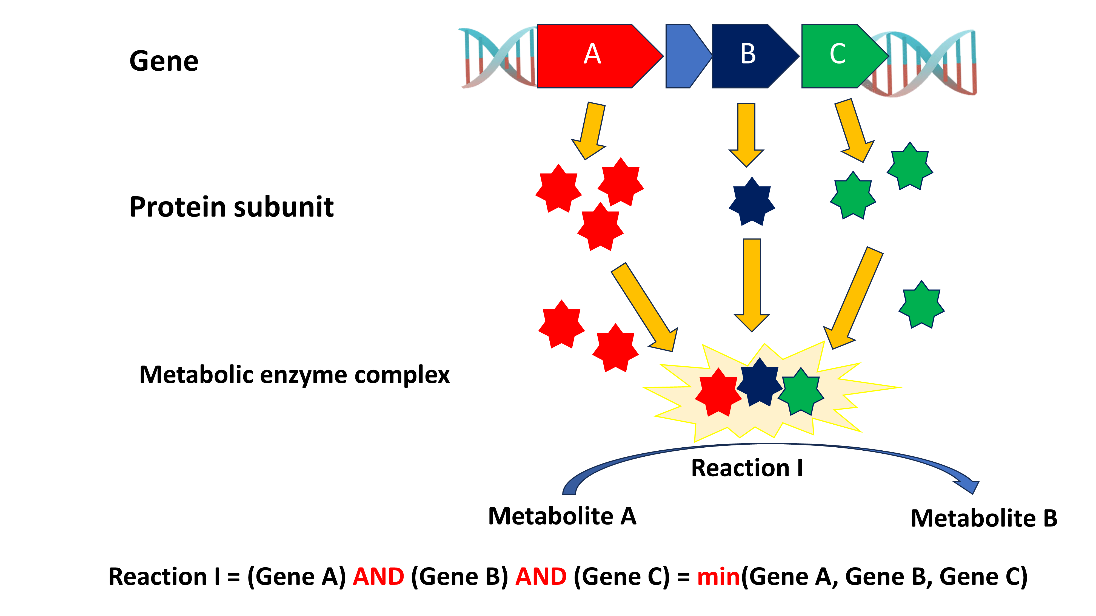


**Figure S1** “AND” relationship in GPR and calculation of gene expression

Gene expression data is incorporated into the metabolic model through the utilization of gene-protein-reaction (GPR) rules. In the context of the "AND" relationship within the GPR rule, refer to Figure S1 for a visual representation. In this framework, a metabolic reaction is influenced by multiple genes responsible for encoding subunits that assemble into a protein complex. The calculation involves determining the minimum value of the associated gene expression levels, following an "AND" format, as the maximum concentration of the enzyme complex is constrained by the minimum concentration of its individual components.

When multiple genes encode isozymes that catalyze a reaction, these isozymes are indicated in a GPR rule using an "OR" format. To determine the total capacity of isozymes for a reaction, we sum the gene expression levels associated with these isozymes. For a more detailed explanation of the "OR" relationship and the calculation of gene expression, please refer to Figure S2.

**
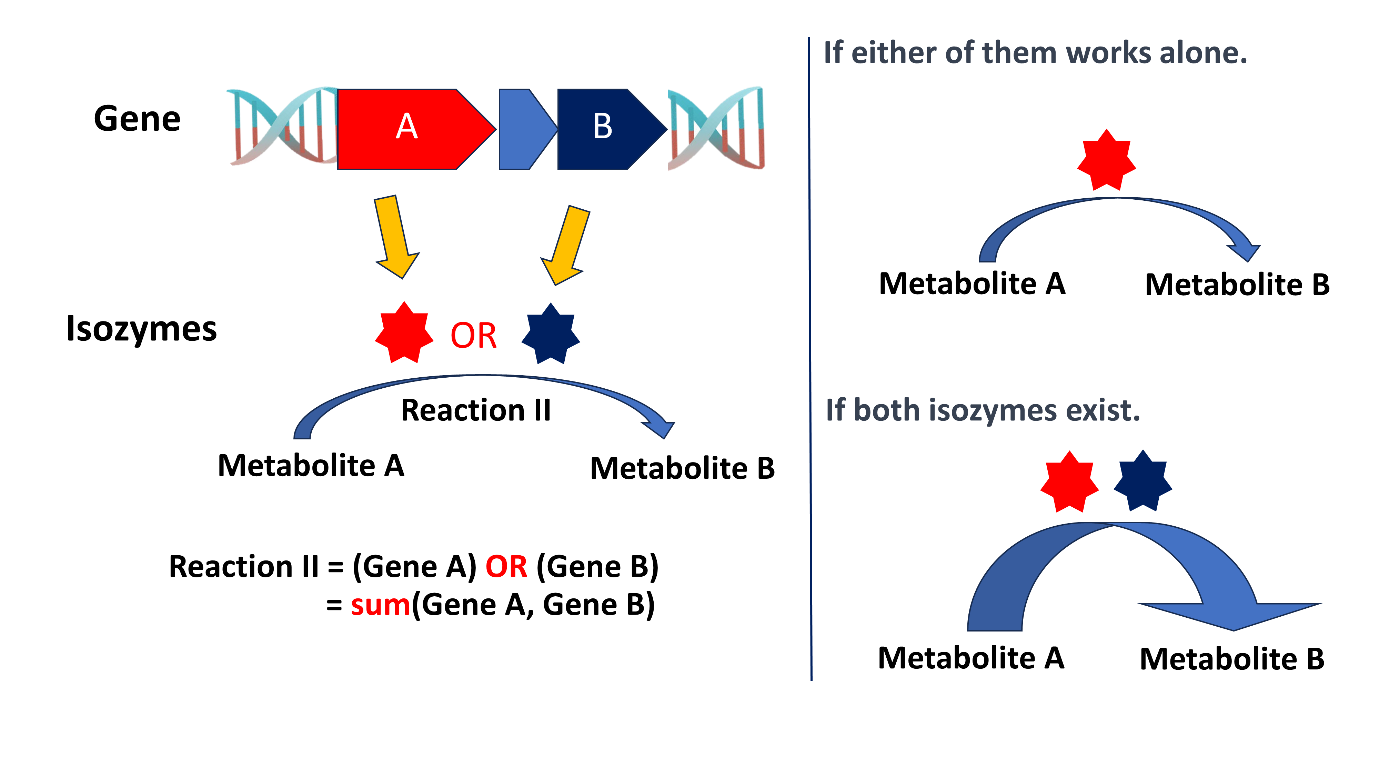
**

**Figure S2** “OR” relationship in GPR and calculation of gene expression

**S5: E-flux method with irreversible reaction flux**

E-Flux is a method for integrating gene expression data into flux balance analysis. It achieves this by establishing the maximum and minimum allowable flux values as a function of the measured gene expression, leveraging the gene-protein-reaction (GPR) associations.

Let $g$ represent the gene expression value corresponding to the metabolic reaction flux. $v^{irr}$denotes a vector of irreversible reaction fluxes, and $v^{rev}$ represents a vector of reversible reaction fluxes.

$Maximize \bar{Z}=\bar{c}^{T}\bar{v}$

Subject to $\bar{S}\bar{v}=0$

$0\leq\bar{v}\leq f(g)$

Where $\bar{c}=\left[ c^{irr} c^{rev} -c^{rev} \right]^{T},\bar{v}=\left[ v^{irr} v^{a} v^{b} \right]^{T}$, and $\bar{S}=[S^{irr} S^{rev} S^{rev}]$.

The $c^{irr}$ and $c^{rev}$ are the vector of $c$ which each component corresponds to the irreversible reaction fluxes, and the irreversible reaction fluxes respectively. $S^{irr}$ and $S^{rev}$ are the submatrix consisting of the column of S corresponding the irreversible reaction fluxes, and the reversible reaction fluxes.

**S6: Quadratic programming for integrating the gene co-expression network into metabolic model based on flux balance analysis**

ICON-GEMs are the quadratic programming for integrating the gene co-expression network into metabolic model based on FBA. The constraints of ICON-GEMs are described below:

$\sum_{j=1}^{n+p} \bar{S}_{ij}\bar{v}_{j}=0$

$0\leq\bar{v}_{j}\leq f(g_{j})$ for all $j=1, 2, 3,\ldots, n+p$

$\sum_{j=1}^{n+p} \bar{c}_{j}\bar{v}_{j}\geq\alpha z^{*}$

$\sum_{(i,j)\in Rev} \bar{v}_{i}\bar{v}_{j}=0$

$\frac{\bar{v}_{j}}{M_{j}}-q_{j}=-1$ for all $j=1, 2, 3,\ldots, n+p$

Suppose that there are $n$ reactions and $p$ reversible reactions in metabolic network. The first constraint ensures the determination of flux in a state of equilibrium and the second constraint is to set the range of reaction fluxes as the function of gene expression value. These constraints are fundamental in our modeling approach and ensures the conservation of mass and reactants within the system which are the same as constraint in E-flux method.

In the third constraint, it becomes imperative to maintain the metabolic objective, such as biomass production, above a prescribed minimum level. This requirement is crucial for ensuring the viability of a given flux distribution. When applying Flux Balance Analysis (FBA) to the design of bacterial strains, it is a common practice to utilize minimum thresholds ranging from 10% to 30% of the optimal objective flux to guarantee this viability. The fourth constraint states that when we decompose a reversible reaction into its constituent irreversible reaction pairs, the sum of the products of these pairs should always result in zero.

Typically, some flux values are considerably lower, while others are significantly higher. To address this disparity and ensure comparability, it is essential to standardize the flux values, bringing them into a uniform range. This standardization process helps in making meaningful comparisons and analysis. The last constraint serves as the transformation equation within our framework. This equation takes the form of a linear function, and its purpose is to convert the reaction flux into a standardized range. The example of transformation is shown in Figure 3S.

**
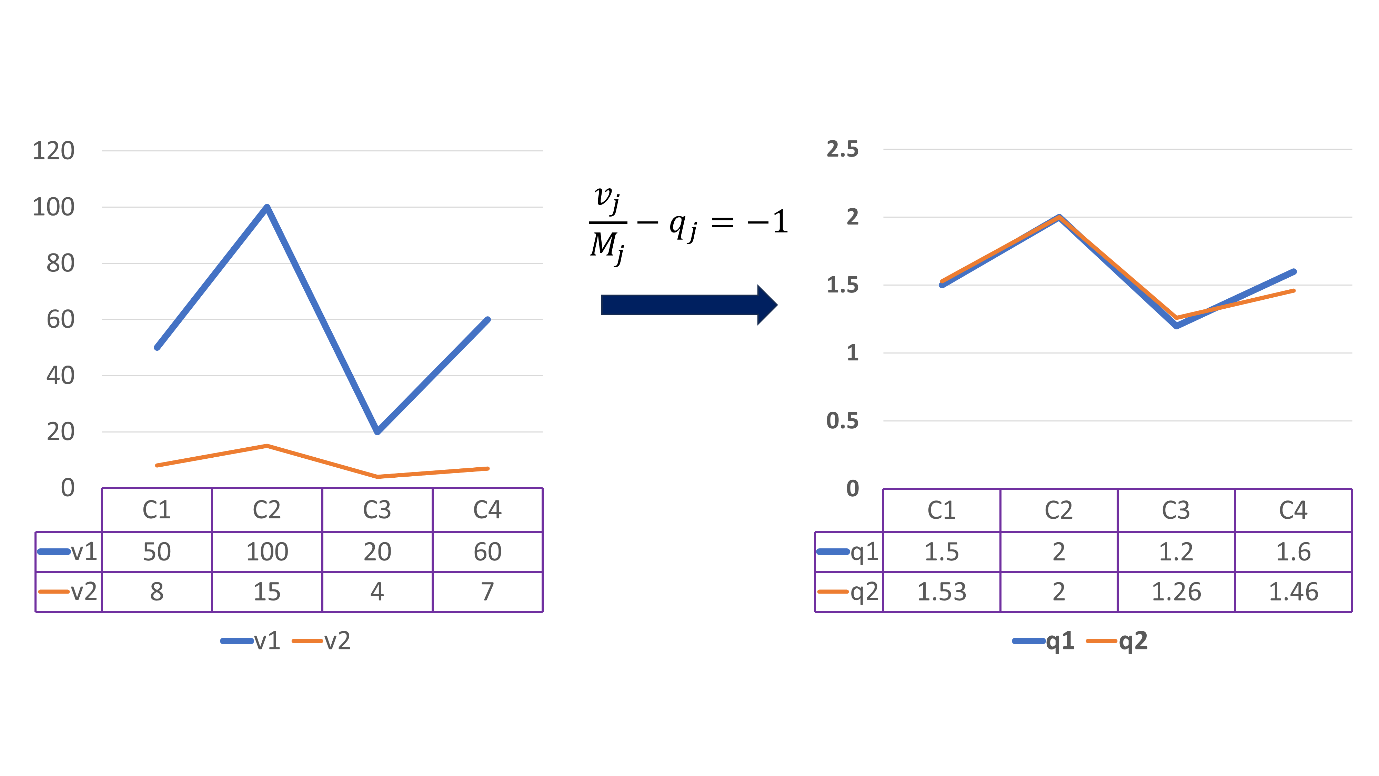
**

**Figure S3** The transformation of reaction fluxes

The objective function in ICON-GEMs aims to maximize a combination of the product of transformed reaction flux pairs using a quadratic programming approach. This maximization process serves to quantify the highest achievable value of the product of these flux pairs, ensuring their consistency. In practical terms, if one of the flux pairs yields a high value, the other pair also exhibits a high value. Conversely, if one of the flux pairs has a low value, the other pair likewise reflects a low value.

Maximize $\sum_{(i,j)\in R} q_{i}q_{j}=q^{T}Aq$

Where $A$ is the symmetry matrix that encodes the relationships between reactions. $A_{ij}=\frac{1}{2}$ when the associated genes of reactions $i$ and $j$ are correlated, and $A_{ij}=0$ otherwise.

**S7: Integration of gene co-expression network into metabolic model framework**

We introduce the rationale for integrating the gene co-expression network and demonstrate how our model works through a small simulation. Let's assume we have gene expression data for three conditions, which we have mapped to three reactions as shown in Table S1. Next, we can determine the bounds of the $q$ values using a linear transformation, as described in the previous section. The $q$ values fall within the range [1, 2]. For instance, for $f(g_{R0})$ in condition c1, we find the range of $q_{R0}$ using the equation $\frac{\bar{v}_{R0}}{M_{R0}}-q_{R0}=-1$, where $M_{R0}=20$ (the highest gene expression value among all three conditions). If we let $\bar{v}_{R0}=0$ (the least possible flux value), we obtain $q_{R0}=1$. Similarly, if $\bar{v}_{R0}=10$ (the maximum possible flux value in condition c1), we calculate $q_{R0}$ to be 1.15. The ranges of $q$ for other reactions or conditions can be found in the Table S1.

**Table S1**: The mapped of gene expression level **Table S2** The range of $q$

on each reaction fluxes.

|  | **c1** | **c2** | **c3** |
| --- | --- | --- | --- |
| $f(g_{R0})$ | **10** | **15** | **20** |
| $f(g_{R1})$ | **5** | **10** | **15** |
| $f(g_{R6})$ | **2** | **4** | **6** |

|  | c1 | c2 | c3 |
| --- | --- | --- | --- |
| $q_{R0}$ | **[1,1.5]** | **[1,1.75]** | **[1,2]** |
| $q_{R1}$ | **[1,1.33]** | **[1,1.67]** | **[1,2]** |
| $q_{R6}$ | **[1,1.33]** | **[1,1.67]** | **[1,2]** |


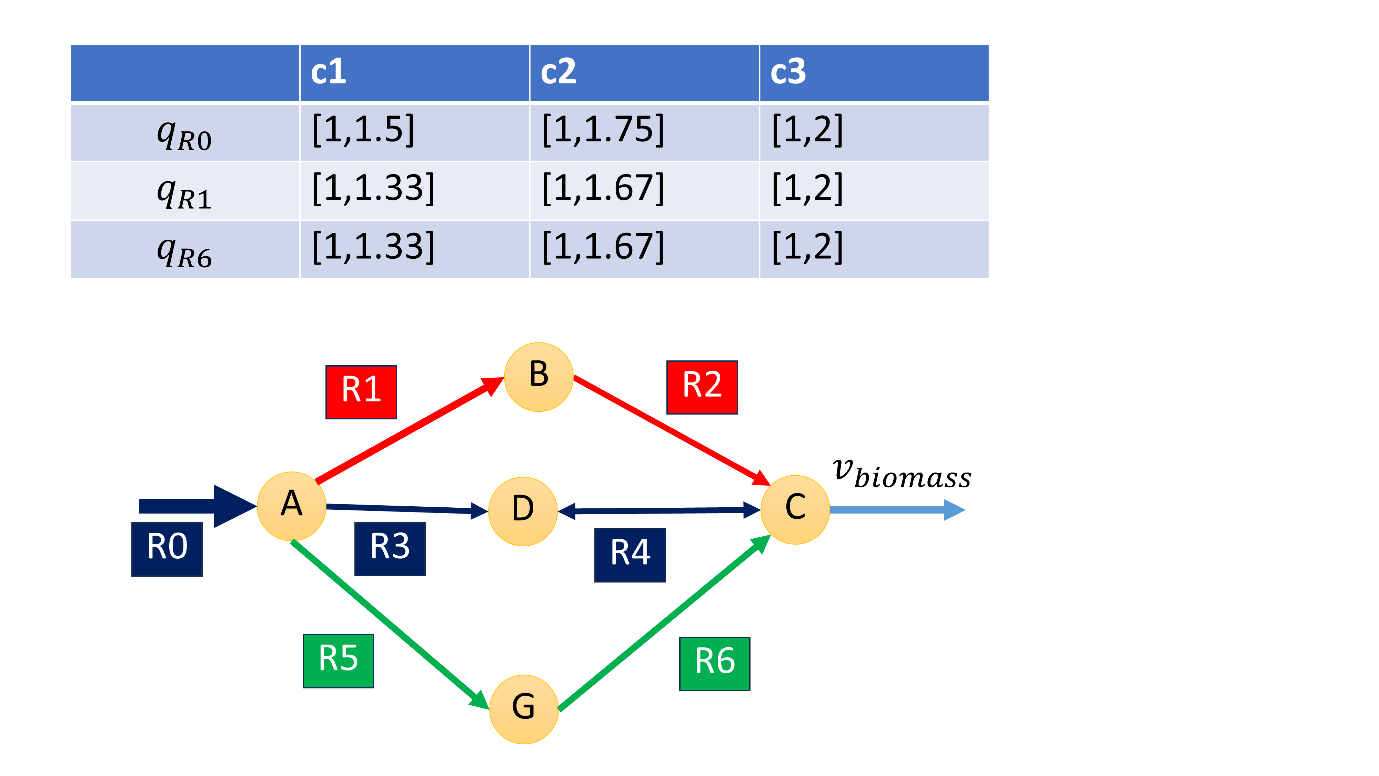


**Figure S4** The simple metabolic network

What we aim to demonstrate next is the use of a quadratic objective function to illustrate the relationship between $q_{i}$ and $q_{j}$, where $i$ and $j$ correspond to the reactions. As depicted in Figure S6, let's assume that the genes involved in reaction R0 are related to the genes involved in reaction R1. In this case, the objective function becomes $\max q_{R0}q_{R1}$, which means that the model prioritizes the flow of the metabolite to pass through reaction R1 before it moves to another path. What we find is that this approach results in consistent flux values across different conditions, as shown in the following table.

|  | C1 | C2 | C3 |
| --- | --- | --- | --- |
| $q_{R0}$ | 1.5 | 1.75 | 2 |
| $q_{R1}$ | 1.33 | 1.67 | 2 |

**Table S3** The calculated q value

By examining these relationships, we can observe that when q values are related, the corresponding flux values also exhibit a relationship based on the linear conversion function used. Similarly, if we consider that the genes involved in reaction R1 are related to those in reaction R6, the model seeks to maximize the values of fluxes for R1 and R6. By doing so, the model aligns the fluxes of these reactions as maximum as possible.

Similarly, if we assume that genes R0, R1, and R6 are related, we can define an objective function as the sum of products between pairs of fluxes. This objective function can be expressed as $\max q_{R0}q_{R1}+q_{R1}q_{R6}$, which means that the model prioritizes the flow of reactions by initially increasing R1 as much as possible and then subsequently increasing R6. In a simple model, these calculations result in consistent flux values.

However, in larger and more complex networks, this process can become intricate. Therefore, a simplified diagram may not suffice to comprehensively explain all scenarios. This complexity can sometimes lead to discrepancies between predicted flux values and real-world observations. Addressing this challenge presents an avenue for future development.

**S8: ICON-GEMs on simple metabolic network**

We assume that genes associated with the same pathway (having similar expression patterns or functional roles) are connected in the gene co-expression network. If two genes are connected in the gene co-expression network, the reactions associated with these genes are also considered connected at the reaction association level. This concept is illustrated in Figure S6.

In Figure S7, we simulate a toy metabolic model with gene expression levels mapped to each reaction. The thickness of arrow edges represents the magnitude of gene expression. When we apply the E-flux method by maximizing the biomass flux, several inconsistencies arise. For example, in the red pathway, the flux distribution matches the minimum gene expression level for that pathway. However, in the blue pathway, the flux distribution contradicts the high gene expression level for reaction R4, resulting in higher flux than reactions R5 and R6, which have lower expression. A similar discrepancy is observed in the green pathway, where reactions R7 and R9 produce lower fluxes despite their gene expression levels. Furthermore, production does not occur as expected.

To address these issues, we propose incorporating gene relationships into the metabolic model to quantify flux distribution. In Figure S7, flux R4 is associated with R5, R6, and R7. With ICON-GEMs, we utilize the summation of products of associated reaction fluxes. This approach allows the flux of R4 to reach its upper bound, even when R5 and R6 have lower flux values, because R4 is correlated with R7, which has a high correlation. As a result, flux flows through R4, R7, and R8, a behavior that does not occur with the E-flux method.


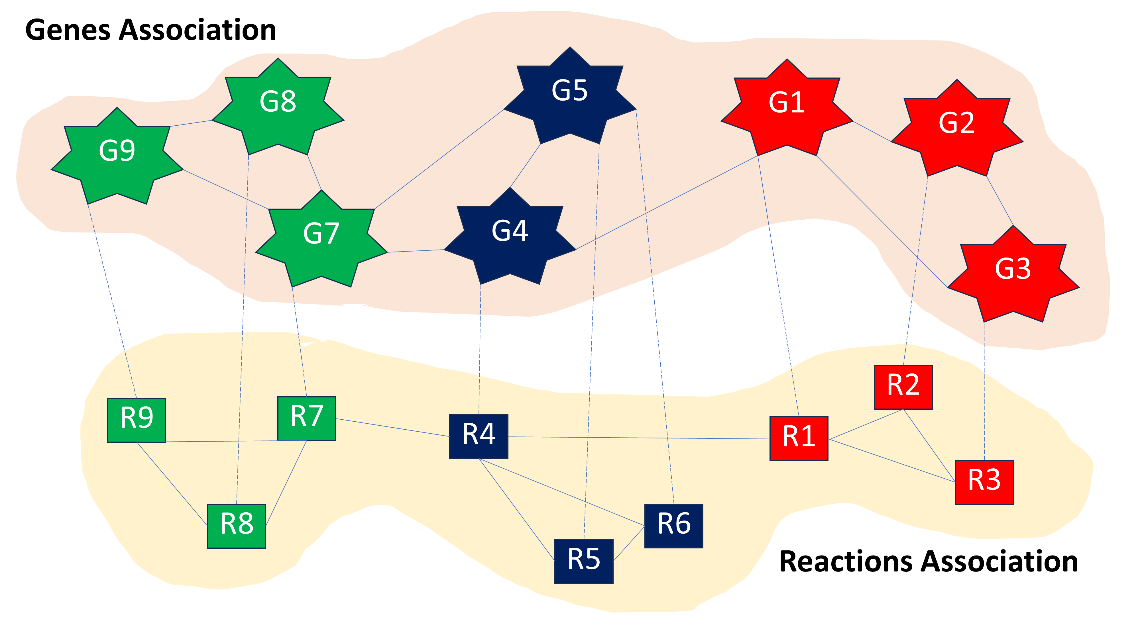


**Figure S5** The example of ICON-GEMs on simple metabolic model

**
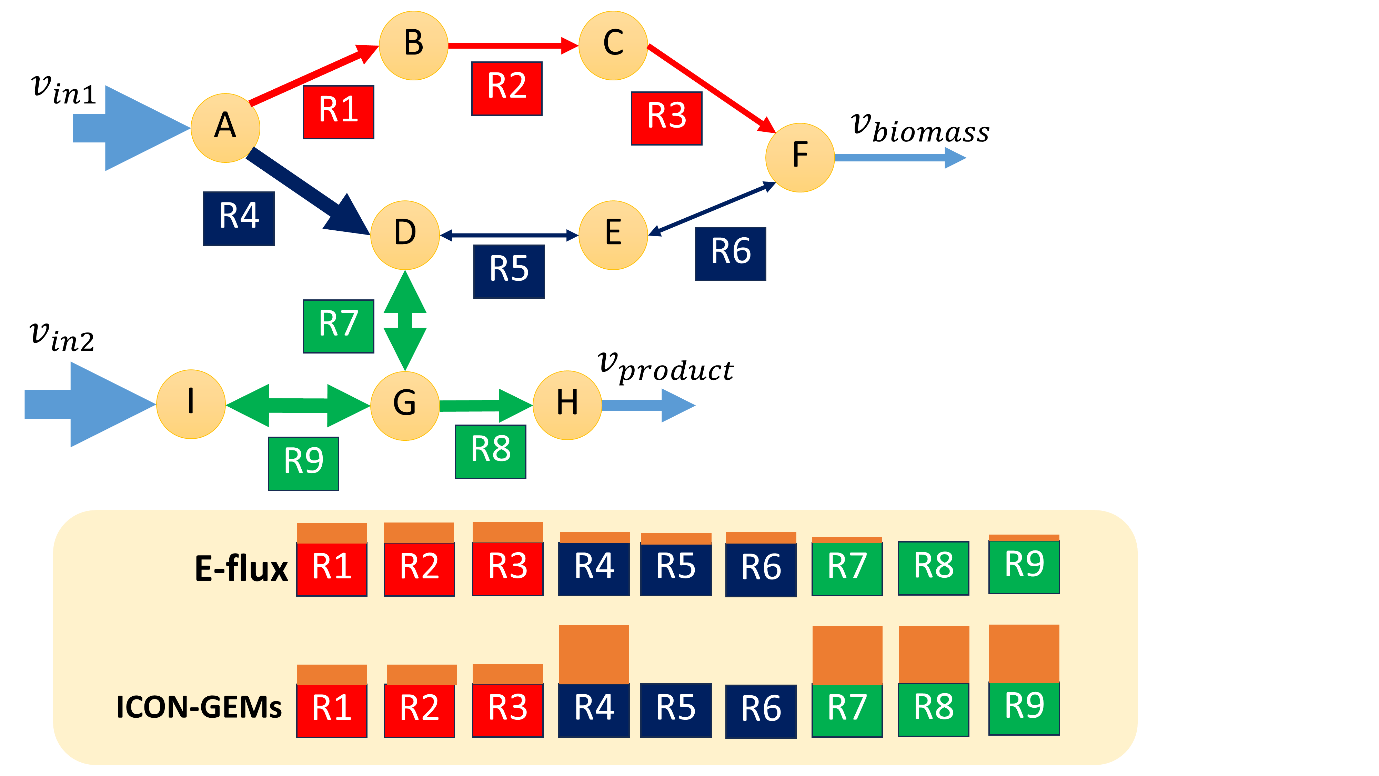
**

**Figure S6** example results of e-flux and ICON-GEMs on simple metabolic network

**S7: The mapping of predicted fluxes and measured fluxes**

Reactions that are experimentally measured and associated with intermediate metabolites are denoted by an "AND" relationship. In our analysis, we determine the minimum flux among these measured reactions. This choice is based on the principle that when multiple measured reactions are linked to the same metabolic pathway as a predicted reaction, the rate of the predicted reaction is constrained by the slowest rate among these measured reactions. The example of “AND” relationship of predicted flux is shown in Table S1.

**Table S1** The example of “AND” relationship and predicted flux calculation

| **Predicted flux** | | **Measured flux** |
| --- | --- | --- |
| **Reaction name** | **Reaction equation** |  |
| R1 | **A** 🡪 B+C | A 🡪 D |
| R2 | B+C 🡪 **D** |  |

From Table S1, the rule for the flux 'A→ D' is expressed as the logical operation (R1) AND (R2). In our analysis, the predicted flux for this reaction is determined by taking the minimum value between R1 and R2. This approach ensures that the predicted flux is bounded by the slower of the two measured reaction rates (R1 and R2) and adheres to the logical relationship described.

The summation of predicted fluxes is computed when a measured flux is associated with multiple predicted reaction fluxes, following an "OR" relationship. This is based on the premise that an increase in the number of reactions capable of carrying out the same chemical conversion may lead to a collective impact on the measured flux. An illustrative example of this "OR" relationship and the corresponding calculation of predicted flux can be found in Table S2.

**Table S2** The example of “OR” relationship and predicted flux calculation

| **Predicted flux** | | **Measured flux** |
| --- | --- | --- |
| **Reaction name** | **Reaction equation** |  |
| **R3** | **A**+B 🡪 C+D+**E** | A 🡪 E |
| **R4** | **A**+F 🡪 G+**E** |  |
| **R5** | **A**+H 🡪 I+**E** |  |

From Table S2, the rule of the flux 'A → E' is described by the logical operation (R3) OR (R4) OR (R5). The predicted flux for this measured reaction flux is calculated as the sum of R3, R4, and R5.
